# Mechanism‐Informed Language Modeling and Oxygenated 3D Screening Identify Berberine–Enzalutamide Synergy in Prostate Cancer Models

**DOI:** 10.1002/advs.78186

**Published:** 2026-10-08

**Authors:** Chih‐Hui Lo, Katie Shi, Lina Kafadarian, Alexandra Bermudez, Johnny Diaz, Liam Edwards, Yunqi Hong, Ziyi Chen, Hyeonji Hwang, Weihong Yan, Alan Levinson, Robert Damoiseaux, Cho‐Jui Hsieh, Tanya Stoyanova, Andrew S. Goldstein, Neil Y.C. Lin

**Affiliations:** ^1^ Bioengineering Department University of California Los Angeles Los Angeles California USA; ^2^ College of Pharmacy National Defense Medical University Taipei Taiwan; ^3^ Mechanical and Aerospace Engineering Department University of California Los Angeles Los Angeles California USA; ^4^ Department of Molecular Cell, and Developmental Biology University of California Los Angeles Los Angeles California USA; ^5^ Computer Science Department University of California Los Angeles Los Angeles California USA; ^6^ Department of Molecular and Medical Pharmacology University of California Los Angeles Los Angeles California USA; ^7^ Department of Chemistry and Biochemistry University of California Los Angeles California USA; ^8^ California NanoSystems Institute University of California Los Angeles Los Angeles California USA; ^9^ Eli and Edythe Broad Center of Regenerative Medicine and Stem Cell Research University of California Los Angeles Los Angeles California USA; ^10^ Jonsson Comprehensive Cancer Center University of California Los Angeles Los Angeles California USA; ^11^ Department of Urology University of California Los Angeles Los Angeles California USA; ^12^ Institute for Quantitative and Computational Biosciences University of California Los Angeles Los Angeles California USA

**Keywords:** 3D spheroids, berberine, drug synergy, enzalutamide resistance, large language models, oxygen supplementation, prostate cancer

## Abstract

Combination therapies are key to overcoming resistance to androgen receptor (AR) signaling inhibitors in prostate cancer. Current paradigms for developing synergistic therapies, however, remain chronically inefficient and resource‐intensive due to the prohibitive scale of the combinatorial screening space and a lack of computational frameworks for navigating signaling crosstalk. This work introduces a hybrid in silico and in vitro lead discovery platform that integrates knowledge‐augmented large language models (LLMs) with an oxygen‐supplemented 3D spheroid system. By comparing compound mechanism‐of‐action annotations with disease‐relevant signaling crosstalk, the LLM framework nominates drug pairs with predictive performance and interpretable, pathway‐based rationales. This computational pipeline is complemented by an engineered 3D spheroid model that utilizes oxygen supplementation to mitigate artifactual necrosis, a common confounder that masks synergistic signals in standard screening. Using this approach to screen 3,592 natural products, we identified berberine‐enzalutamide as an in vitro combination hit that re‐sensitized resistant prostate cancer cells to AR blockade. Molecular and transcriptomic profiling revealed changes in mTORC1 and AMPK signaling that were consistent with the pathway‐based hypothesis generated by LLM. These results establish proof of concept that mechanism‐informed language modeling coupled with 3D screening can prioritize and experimentally evaluate drug‐combination hypotheses at an early stage of discovery.

## Graphical abstract

By integrating knowledge‐augmented artificial intelligence with an oxygen‐supplemented 3D screening platform, a synergistic interaction between berberine and enzalutamide is identified. The framework integrates compound mechanisms of action with disease‐relevant signaling context to prioritize candidate combinations and generate testable pathway‐based rationales, providing a scalable and transparent methodology for the discovery of candidate combination therapies in treatment‐resistant prostate cancer.

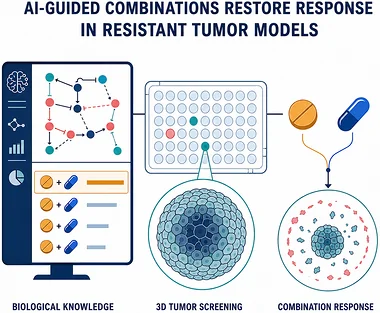

## 1 Introduction

Prostate cancer remains a leading cause of male cancer mortality globally [1]. Its standard of care, androgen deprivation therapy, exhibits only transient clinical efficacy, as most tumors eventually evolve into a castration‐resistant state [2]. In this state, next‐generation androgen receptor (AR) signaling inhibitors, such as enzalutamide, are prescribed. Despite a notable improvement in disease control in the initial phase of treatment, clinical efficacy is short‐lived due to the rapid emergence of acquired resistance [3, 4, 5]. This resistance is driven by a highly plastic landscape where cancer cells activate compensatory signaling as a survival mechanism to bypass AR inhibition [6, 7, 8, 9, 10, 11, 12]. Therefore, exploring synergistic drug combinations that can co‐inhibit these bypass mechanisms is essential to overcome resistance and improve patient outcomes [11, 13, 14]. Unfortunately, the high‐dimensional nature of the combinatorial search space makes exhaustive experimental screening infeasible, even with advanced high‐throughput automation.

To address this challenge, in silico models have been integrated into screening pipelines as predictive filters for prioritizing drug combinations. Supervised deep learning frameworks, including MatchMaker and DeepDDS, learn associations between labeled synergy outcomes and structured chemical or cell‐line features [15, 16]. Related approaches have incorporated pathway information, molecular networks, and multimodal biological data to improve predictive performance and interpretability [17, 18, 19, 20, 21]. Nevertheless, these models generally require substantial labeled training data, and their predictions may provide limited pathway‐level rationale for how a candidate combination interacts with a disease‐specific signaling network. Recent studies have therefore explored large language models (LLMs) for drug‐synergy prediction in data‐limited settings. CancerGPT leverages pretrained language representations and external synergy data for few‐shot transfer across cancer tissues [22], whereas SynerGPT uses in‐context learning from small sets of context‐specific synergy observations [23]. Although these approaches demonstrate the potential of LLMs to support drug‐combination prediction, stochastic generation and unsupported biological statements remain important concerns [24, 25, 26, 27]. Reliable application of LLMs to drug prioritization therefore requires both explicit characterization of prediction stability and independent evaluation of the factual support underlying their biological context.

While computational models offer powerful tools for candidate prioritization, experimental screening remains essential for clinical translation. Standard two‐dimensional (2D) monolayer cultures are highly scalable, but they fail to recapitulate the complex spatial architecture and cell–cell interactions that drive drug resistance in vivo [28]. Three‐dimensional (3D) spheroids provide a more physiologically relevant microenvironment; however, their broader utility is hindered by physical constraint‐induced necrosis. As spheroids exceed 400–500 μm, diffusion‐limited oxygen gradients drive the formation of stagnant necrotic cores [29, 30, 31, 32, 33]. Although central necrosis is a hallmark of hypoxic tumors in vivo, the excessive, uncontrolled cell death and disrupted cell junctions observed in 3D models act as profound confounding factors. This artifactual necrosis obscures specific signaling responses, causing drug‐response measurements to reflect model‐induced stress rather than true therapeutic sensitivity [32, 33, 34, 35, 36].

Here, we present a discovery‐validation framework centered on MAESTRO (Mechanistic Augmentation and Ensemble Selection for Therapeutic Ranking Optimization). In this name, “Mechanistic Augmentation” refers to conditioning the LLM on literature‐supported compound targets and effects, downstream pathway relationships, and curated disease‐specific signaling crosstalk. MAESTRO integrates this knowledge to rank candidate combinations and generate testable, mechanism‐informed hypotheses rather than infer causal mechanisms de novo. Prostate cancer was selected as the initial test case because resistance to AR inhibition involves well‐characterized compensatory signaling crosstalk, while models with distinct AR states and enzalutamide sensitivities enabled prospective validation across defined biological backgrounds. We coupled MAESTRO with an oxygen‐supplemented 3D spheroid platform to test prioritized combination hypotheses under conditions that reduce hypoxia‐associated confounding. Using this workflow, we identified a previously unrecognized in vitro interaction between berberine and enzalutamide. Molecular and transcriptomic profiling showed changes in mTORC1 and AMPK signaling that were consistent with the rationale generated by MAESTRO. Thus, the present study demonstrates MAESTRO's utility as an early‐discovery framework for prioritizing candidate combinations and experimentally evaluating pathway‐based hypotheses, providing a scalable path from mechanism‐informed prediction to in vitro validation.

## 2 Results

### 2.1 Knowledge Augmentation and Ensemble Consensus Enable High‐Confidence Synergy Prioritization

The MAESTRO framework (Figure 1a) is a knowledge‐augmented prioritization pipeline that integrates compound mechanism‐of‐action (MoA) annotations with a curated, disease‐specific signaling context to rank candidate combinations and generate pathway‐based rationales. Central to this approach is the direct integration of pre‐collected biochemical data into the model's context. Leveraging the advanced natural language processing capabilities of LLMs, we systematically synthesize two molecularly annotated textual datasets into context‐augmented prompts: (i) compound‐specific profiles detailing primary and secondary MoA, and (ii) a concentrated, disease‐specific knowledge base distilled from curated literature reviews. This context is structured to capture essential biological dimensions, including AR signaling crosstalk, acquired resistance mechanisms, and upstream regulatory networks that drive adaptive survival. By integrating these distinct mechanistic features, MAESTRO provides the LLM with literature‐curated context to prioritize therapeutic candidates based on their potential for orthogonal inhibition of bypass pathways. This architecture reframes the discovery process from a correlational search into a mechanism‐informed rank‐ordering of potential synergies.

**FIGURE 1:**
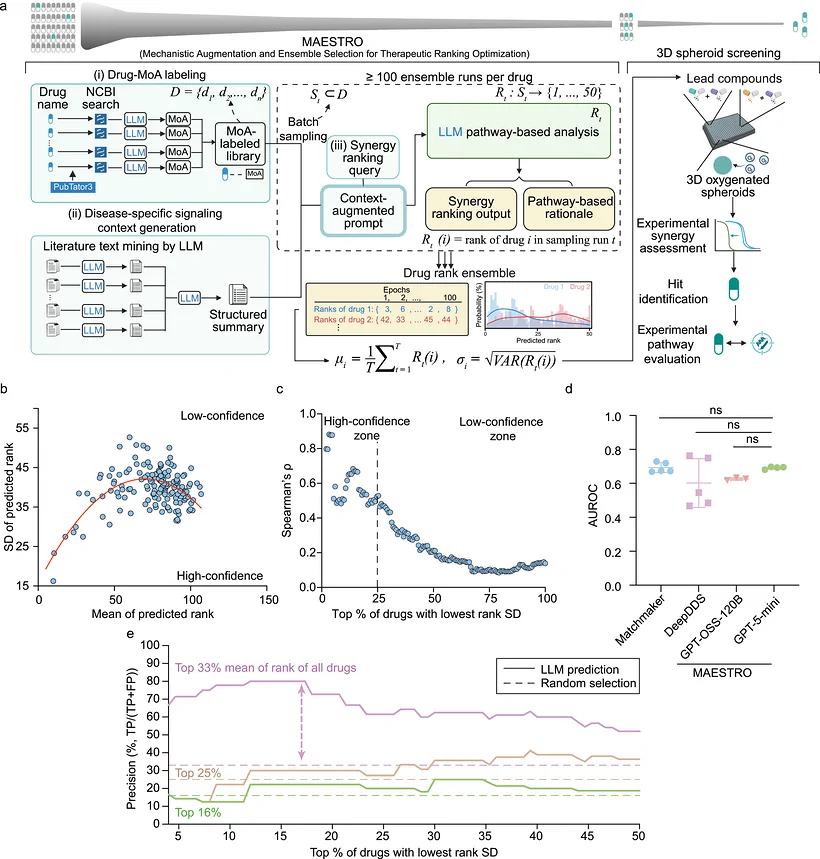
MAESTRO integrates Mechanistic Augmentation with ensemble sampling for drug‐combination prioritization. (a) To discover lead compounds, the MAESTRO pipeline begins by generating (i) LLM‐derived compound mechanism‐of‐action (MoA) labels grounded in NCBI research articles. It also generates (ii) disease‐specific contexts mined from the literature. Utilizing randomized batch sampling, subsets of compound labels are iteratively combined with the disease‐specific context and (iii) a synergy‐ranking query to construct context‐augmented prompts as input for LLM inference. These prompts are processed by LLMs to generate rank‐based synergy predictions along with pathway‐based rationales. This process is repeated until a minimum of 100 ensemble runs is accumulated for each compound. The mean rank summarizes consensus prioritization, whereas the rank standard deviation (SD) quantifies sampling variability. High‐confidence lead candidates are finally validated in an oxygen‐supplemented 3D prostate cancer spheroid model. D, whole compound library; $S_{t}$, randomly sampled subset at iteration t; $R_{t}\left(i\right)$, rank of compound i at iteration t. (b) Scatter plot illustrating the inverted parabolic relationship between MAESTRO‐predicted mean rank and rank standard deviation (SD) for 150 AR inhibitor‐anchored drug pairs from the DrugComb database (400 runs per compound). Low rank SD signifies high‐confidence predictions, whereas high rank SD denotes low confidence. The red line represents a second‐order polynomial fit. (c) Spearman's rank correlation (ρ) between predicted and experimental rankings versus the percentage of drugs included, sorted by ascending rank SD. (d) Scatter dot plot showing the AUROC values for MatchMaker (n=5), DeepDDS (n=5), and MAESTRO (GPT‐OSS‐120B, n=3; GPT‐5‐mini, n=4) when evaluated against Loewe synergy scores. Testing was performed on a subset of 89 abiraterone‐anchored combinations in PC‐3 cells. Lines are presented as mean ± SD. Statistical significance was determined using one‐way ANOVA followed by Dunnett's multiple comparisons test. ns: not significant (p>0.05). (e) Prediction precision profiles plotted against the cumulative percentage of drug pairs included, ordered by ascending rank SD. Solid lines represent precision for varying candidate selection thresholds (top 16%–33% based on mean rank), while horizontal dashed lines indicate the corresponding expected precision under random selection. TP, true positive; FP, false positive.

To ensure the factual accuracy of the knowledge provided to the LLM for prediction and reasoning, we separately audited the MoA annotations supplied to MAESTRO. Forty natural products were randomly selected, and paired annotations generated with and without RAG were evaluated in a blinded, claim‐level review. RAG increased the number of independently verifiable MoA claims while improving the evidence‐supported rate from 78.60% ± 23.41% to 95.11% ± 8.72% (p<0.0001; Figure S1a,b). These findings demonstrate that retrieval augmentation reduces unsupported content in the compound‐specific context used for ranking, although it does not eliminate all epistemic uncertainty in downstream predictions.

We next observed that LLM‐predicted rankings exhibited a degree of stochasticity. Rather than treating stochastic variation across LLM outputs solely as a limitation, we used repeated sampling to derive reproducible ranking statistics, including mean rank and rank variance. Through an ensemble‐ranking framework, we harnessed generative variability to evaluate candidate synergies via aggregated, iterative batch‐wise competitive analyses. We applied this approach to rank 50 candidate compounds for synergy with enzalutamide, utilizing an iterative sampling strategy to define cohorts and mitigate batch‐specific bias. Our results show that mean ranks converged to ∼1% variation after 100 independent runs, yielding a highly stable ranking prediction (Figure S1c). Importantly, for each candidate assessed, the LLM generated a justification for each candidate to support its assigned rank (Figure S1d).

The two quantitative outputs of MAESTRO are (i) a mean rank ($\mu_{i}$) representing the predicted consensus synergistic potential and (ii) a rank standard deviation ($\sigma_{i}$) that quantifies sampling variability across repeated predictions. We benchmarked MAESTRO's performance against 150 AR inhibitor‐anchored drug pairs from the DrugComb database [37]. Our analysis revealed a characteristic “inverted U‐shaped” distribution relating mean rank to variance (Figure 1b), demonstrating that the LLM achieves maximal consensus at the phenotypic extremes of potent synergy and strong antagonism. We defined these low‐variance regimes as the “High‐Confidence Zone” (HCZ), where the model's predictions are most consistent. Within the HCZ, the framework achieved a Spearman correlation of ρ∼0.87 between predicted and experimental rankings (Figure 1c and Figure S1e). Expanding the selection criteria to incorporate high‐variance candidates precipitously attenuated the correlation to ρ∼0.1, supporting rank variance as an empirical indicator of predictive reliability in the evaluated datasets. This mean‐variance relationship and the resulting predictive trends were consistently recapitulated in an independent abiraterone‐anchored PC‐3 dataset (Figure S2a,b) and across alternative LLM architectures (Figure S2c– f). Within the HCZ, rankings generated by the GPT and Gemini backends were correlated (ρ∼0.97), indicating similar prioritization by these two backends (Figure S2e,f). To test whether MAESTRO's predictive framework extends beyond prostate cancer and AR‐directed therapy, we evaluated two additional DrugComb datasets, each comprising 108 5‐fluorouracil‐anchored combinations tested in HCT‐116 or SW‐620 colorectal cancer cells. In both datasets, concordance between predicted and experimental rankings increased as the analysis was restricted to drug pairs with lower rank SD and was greater in the HCZ than in the complete dataset (Figure S2g–i). Thus, the rank variance‐predictive accuracy relationship was reproduced with a different cancer type, anchor drug, and cellular context.

To test MAESTRO with open‐weight models, we repeated the benchmark analyses using GPT‐OSS‐20B and GPT‐OSS‐120B. Both models reproduced the characteristic mean rank–variance relationship and showed the strongest agreement with experimental rankings among low‐SD candidates in the AR inhibitor‐anchored and abiraterone‐PC‐3 datasets, respectively (Figure S3a– c). We further assessed cross‐backend concordance using three independently sampled sets of 700 natural products. Rankings from GPT‐OSS‐120B, GPT‐OSS‐20B, and Qwen3.5‐35B‐A3B correlated strongly with GPT‐4o rankings (mean Spearman's ρ > 0.70), and each recovered the GPT‐4o‐defined top 5% with an AUROC > 0.90 (Figure S3d– f). Thus, MAESTRO's uncertainty structure and top‐candidate prioritization were reproducible across proprietary and open‐weight backends.

To establish MAESTRO's relative efficacy, we benchmarked its zero‐shot performance against two representative deep learning architectures: MatchMaker and DeepDDS. Evaluated on the aforementioned abiraterone‐anchored dataset, MAESTRO achieved predictive accuracy within the range observed for these conventional models, despite them requiring “leave‐drug‐and‐cell‐out” training on the DrugComb database. Specifically, MAESTRO demonstrated on‐par AUROCs for Loewe scores (Figure 1d) and Bliss scores (Figure S4a) relative to both trained baselines. Within its HCZ, MAESTRO's Spearman correlation matched MatchMaker and exceeded DeepDDS (Figure S4b). Crucially, MAESTRO achieves this quantitative accuracy while simultaneously generating interpretable justifications for its predictions.

Lastly, we interrogated two key architectural parameters to elucidate the primary performance determinants of the MAESTRO pipeline. First, we conducted an ablation analysis to quantify the impact of the explicitly provided biochemical context on predictive accuracy. Removing this contextual augmentation substantially degraded performance, reducing the Spearman correlation within the HCZ from 0.86 to 0.43 (Figure S2b). Second, we evaluated how ranking and variance thresholds influence predictive precision. Restricting candidate selection to the top 33% within the most stringent variance window achieved a precision of approximately 70%, a two‐fold enrichment over the random expectation baseline (Figure 1e). These results establish an optimal operational threshold that maximizes lead‐candidate accuracy while preserving the scalability of the screening process.

### 2.2 MAESTRO Prioritizes Candidate Synergistic Combinations Through Knowledge‐Augmented Analysis

To evaluate whether MAESTRO's predictions extend beyond direct literature retrieval, we tested whether it could integrate compound MoAs with supplied signaling relationships to prioritize previously unreported combination hypotheses. We applied the framework to a library of 1,250 protein kinase inhibitors (PKIs) to rank candidates for potential synergy with enzalutamide. This well‐defined pharmacological space allowed us to effectively assess whether the model could integrate signaling crosstalk to prioritize unreported combination hypotheses, rather than merely retrieving documented synergies from literature. The virtual screening recapitulated the characteristic “inverted U‐shaped” mean rank‐uncertainty distribution (Figure 2a), enabling us to prioritize 95 candidates within the HCZ. Although tyrosine kinase inhibitors numerically dominated the baseline library, compounds targeting the PI3K/AKT/mTOR pathway exhibited the highest enrichment rate among the top candidates (15.7%; Figure S5a). We then conducted a systematic literature cross‐validation of these 95 prioritized leads and found that 65.63% lacked any previously reported synergy with enzalutamide (Figure 2b). MAESTRO integrates established compound MoAs with signaling relationships associated with enzalutamide resistance to generate combination‐level hypotheses; it does not claim to discover de novo pathway biology. The validation outcomes were generated prospectively after ranking and were unpublished when the predictions were made, thereby providing evaluation on previously unavailable pair‐level outcomes. A prespecified literature search further identified no previous report of enzalutamide synergy for more than 65% of the prioritized PKI pairs, although undisclosed pretraining corpora prevent complete exclusion of prior exposure to pair‐specific literature.

**FIGURE 2:**
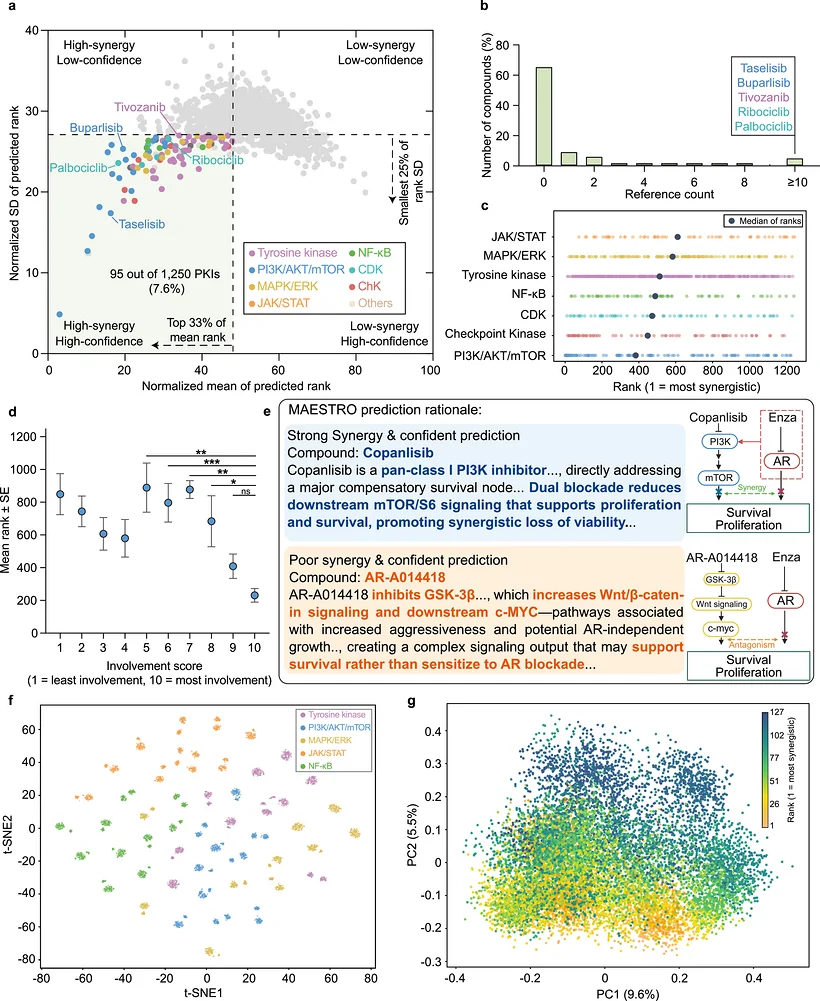
MAESTRO generates pathway‐aligned rationales for kinase inhibitor prioritization. (a) Scatter plot of MAESTRO‐predicted normalized standard deviation of predicted rank versus normalized mean of predicted rank for 1250 PKIs (400 runs per compound). Selection of 95 prioritized candidates was defined by a top 33% cutoff for mean rank and a smallest 25% cutoff for rank SD. (b) Frequency of publications reporting synergy between enzalutamide and each of the 95 lead compounds. Data were obtained via manual curation of Google Scholar. (c) Distribution of predicted synergy ranks across seven mechanism‐based categories, with median ranks indicated by a black dot for each category. Low rank value corresponds to high synergy prediction. (d) Mean predicted synergy rank plotted against involvement score for 127 inhibitors within the PI3K/AKT/mTOR pathway. This LLM‐derived score (1–10) quantifies the directness of targeting key pathway nodes, where higher values signify core pathway inhibition. Data are presented as mean ± SE. (e) Representative MAESTRO‐generated prediction rationales accompanied by schematic illustrations comparing high‐ (blue) and low‐synergy (yellow) candidates within the PI3K/AKT/mTOR pathway category. The red box in the inset highlights AR inhibition, which triggers a compensatory signaling pathway. (f) t‐SNE visualization of semantic embeddings derived from generated rationales across five categories (10 compounds per category; 100 rationales per compound). (g) PCA plot of rationale embeddings for the PI3K/AKT/mTOR‐associated category (127 compounds; 100 rationales per compound). Data points are colored by predicted synergy rank. Statistical significance was determined by one‐way ANOVA followed by Dunnett's multiple comparisons test; *p<0.05, **p<0.01, ***p<0.001, ****p<0.0001, ns: not significant.

We then sought to examine pathway‐level patterns associated with MAESTRO‐derived rankings. We first characterized the macro‐level features associated with MAESTRO‐derived rankings. An inter‐pathway analysis of the 1,250 PKIs revealed shifts in median rank distributions across categories, confirming that the model incorporates pathway‐level selectivity into its predictive logic (Figure 2c). Moreover, the substantial intra‐pathway variance indicates that MAESTRO discriminates among individual molecular profiles within the same functional class. To dissect the sources of this intra‐group variation, we analyzed target involvement within the top‐ranked PI3K/AKT/mTOR pathway to characterize semantic patterns within the generated rationales. Specifically, an LLM‐derived involvement score (1–10) was applied to each of the 127 inhibitors to estimate their directness in targeting key signaling nodes. This analysis demonstrated a strong correlation between involvement scores and MAESTRO rankings, in which higher scores (indicating close proximity to the pathway core) aligned with greater predicted synergy (Figure 2d). This discrimination is exemplified by the contrasting prioritizations of Copanlisib and AR‐A014418 (Figure 2e). Copanlisib, recognized as a core PI3K/AKT/mTOR pathway inhibitor, achieved a high involvement score and was correspondingly prioritized for its capacity to abrogate compensatory survival signaling. Conversely, AR‐A014418 yielded a low involvement score and a commensurately poor ranking due to its targeting of peripheral nodes and an elevated potential for antagonism. MAESTRO's textual justifications demonstrate a prioritization of core pathway hubs over functionally distal nodes.

To validate the alignment between numerical rankings and its text‐based justifications, we performed an embedding‐based semantic analysis of the LLM‐generated rationales. At the inter‐pathway level, t‐SNE visualization revealed that rationales for distinct compound classes segregated into well‐defined semantic clusters, indicating that the model's reasoning is consistent and functionally compartmentalized (Figure 2f). To decode the intra‐pathway prioritization logic, we performed principal component analysis (PCA) exclusively on the PI3K/AKT/mTOR rationales. This revealed a continuous semantic gradient along the PC2 axis that aligned with numerical rankings (Figure 2g). This relationship was corroborated by token frequency analysis (Figure S5b), which showed that high‐priority rationales were enriched with terms governing core pathway modulation (e.g., “mTOR,” “dual blockade,” “compensatory”), whereas low‐priority rationales were dominated by terminology reflecting peripheral node interactions. Collectively, these findings confirm that MAESTRO's quantitative predictions are coupled to a coherent, context‐aware framework.

### 2.3 Oxygen Supplementation Reduces Necrotic‐Core Interference in 3D Prostate Cancer Spheroids

Having established MAESTRO's capacity to distill the candidate drug space, we transitioned to experimental validation of the nominated synergies using 3D prostate cancer spheroids (Figure 3a). Although 3D models capture spatial features absent from standard 2D cultures, their utility is often constrained by limited oxygen diffusion. Specifically, oxygen limitation can produce substantial central cell death, compressing the dynamic range of viability‐based assays. To circumvent this technical hurdle, we engineer an oxygen‐supplemented platform that mitigates necrotic core formation while maintaining compatibility with standard 96‐ and 384‐well screening formats. We validated this platform across a panel of prostate cancer models with distinct sensitivities, including enzalutamide‐sensitive LNCaP and enzalutamide‐resistant 22Rv1 and C4‐2B MDVR (MDVR) [38] cell lines. The objective of oxygen supplementation was not to reproduce the oxygen environment of an intact prostate tumor, but to reduce diffusion‐limited baseline cell death that interferes with viability‐based screening in large avascular spheroids. Because the incubator oxygen concentration does not directly represent the oxygen concentration experienced by cells at different spheroid depths, we empirically evaluated 21%, 40%, 60%, and 95% $O_{2}$ and selected 40% $O_{2}$ as the lowest supplemental condition that substantially reduced central apoptosis without producing detectable oxidative injury (Figure S6a–c).

**FIGURE 3:**
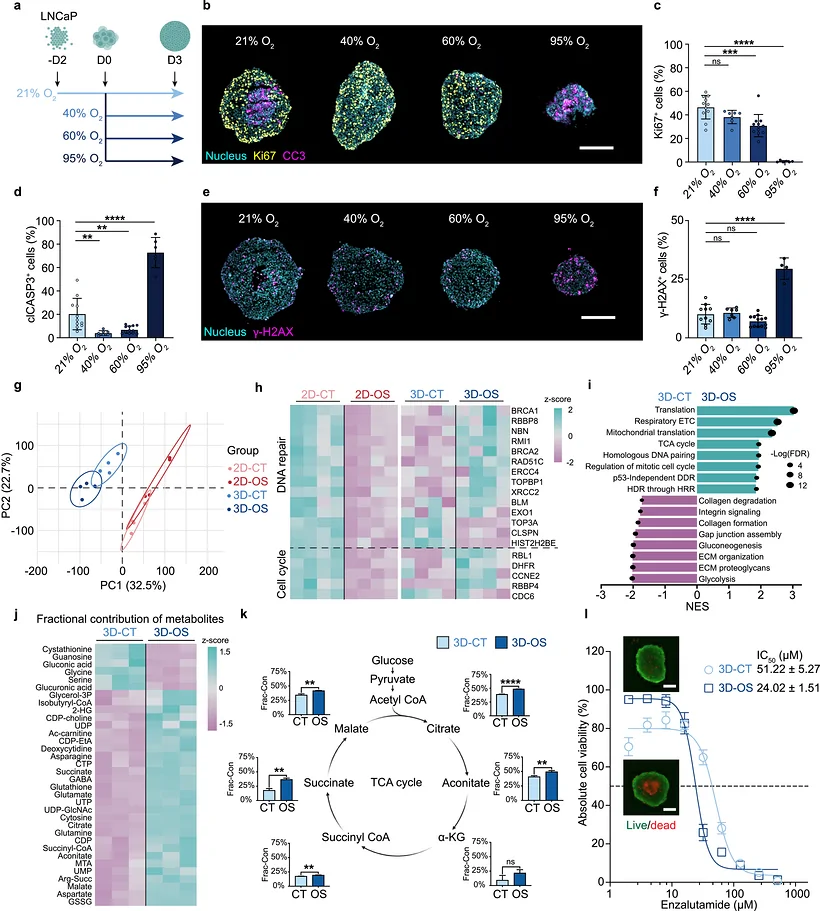
Oxygen supplementation normalizes viability, metabolism, and drug sensitivity in 3D prostate cancer spheroids. (a) Schematic illustration of the oxygen supplementation experiment. LNCaP spheroids were cultured under varying oxygen levels (21%–95% $O_{2}$) to identify the optimal condition for minimal necrotic core formation. (b) Representative immunofluorescence images of cells stained for the proliferation marker Ki67 and the apoptosis marker cleaved caspase‐3 (CC3) following 3 days of oxygen‐supplemented culture. Scale bar, 200 μm. (c, d) Quantification of percentage of (c) Ki67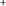 cells and (d) CC3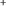 cells under the indicated oxygen levels (n=6−12 per group). (e) Representative immunofluorescence images of γ‐H2AX (DNA damage marker) following 3 days of oxygen‐supplemented culture. Scale bar, 200 μm. (f) Quantification of percentage of γ‐H2${\mathrm{AX}}^{+}$ cells under indicated oxygen levels (n=4−13 per group). (g) PCA plot of RNA‐seq profiles across four culture conditions: 2D 21% $O_{2}$ (2D‐CT), 2D 40% $O_{2}$ (2D‐OS), 3D 21% $O_{2}$ (3D‐CT), and 3D 40% $O_{2}$ (3D‐OS). Ellipses represent 95% confidence ellipses. (h) Heatmap visualizing the z‐scored expression patterns of DNA repair and cell cycle‐related genes across the four groups. (i) Gene set enrichment analysis comparing 3D‐OS versus 3D‐CT. The bar chart displays representative upregulated and downregulated pathways, determined by the normalized enrichment scores (NES). The size of the black dot represents the −log(FDR), where FDR denotes the false discovery rate. Data in (g–i) are derived from n=4 biological replicates. (j) Heatmap of z‐scored fractional contributions of 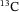
‐glucose labeled metabolites in 3D‐OS and 3D‐CT conditions. (k) Bar charts depicting fractional contributions of TCA cycle intermediates comparing 3D‐OS and 3D‐CT. The middle schematic illustrates the TCA cycle. Data in (j–k) are derived from n=3 biological replicates. (l) Enzalutamide dose‐response curves plotting absolute viability derived from Live/Dead staining for LNCaP spheroids in 3D‐CT and 3D‐OS groups (n=4). Representative images of untreated controls at Day 3 are shown as insets. Scale bar, 200 μm. Data are presented as mean ± SD. Statistical significance was determined by one‐way ANOVA followed by Dunnett's multiple comparisons test (c, d, f) or two‐tailed unpaired Student's *t*‐test (k); * p<0.05, ** p<0.01, *** p<0.001, **** p<0.0001, ns: not significant.

Spatial analysis showed that the reduction in dead cells and cleaved caspase‐3 (CC3; apoptosis biomarker) at 40% $O_{2}$ occurred predominantly within the spheroid core. Ki‐67 (proliferation biomarker), cleaved caspase‐3, and E‐cadherin (cell‐cell junction biomarker) measurements in the non‐core compartments did not reveal increased proliferation, apoptosis, or loss of epithelial organization (Figure 3b–d and Figures S6d,e and S7a–c). In addition, 40% $O_{2}$ did not significantly increase whole‐spheroid ROS or γ‐H2AX (DNA double‐strand breaks biomarker) or reduce prostate‐specific antigen (PSA) production (Figure 3e–f and Figure S7d– f). By contrast, 95% $O_{2}$ markedly increased oxidative stress and DNA damage, providing a positive control for oxygen‐induced cellular injury.

Omics analyses further corroborated the absence of substantial hyperoxic stress. Transcriptomic profiling showed that the DNA repair and cell cycle suppression typically induced by 40% $O_{2}$ in 2D monolayers were abrogated in 3D spheroids. This attenuated stress signature indicates that the 3D spatial architecture buffers the tissue against hyperoxia‐induced toxicity (Figure 3g–i and Figure S8a– c). Concurrent with this lack of stress signaling, gene set enrichment analysis (GSEA) and 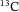
‐glucose metabolic tracing revealed that 40% $O_{2}$ enhanced glucose incorporation into the TCA cycle and increased the relative abundance of TCA cycle‐related metabolites (Figure 3i–k and Figure S9a– b). Crucially, comparative metabolic profiling against 21% $O_{2}$ controls confirmed that this oxygenation strategy did not induce broad reprogramming of classical prostate cancer metabolic axes, leaving glycolytic lactate production and the pentose phosphate pathway unperturbed (Figure S9c– f). Together, these findings suggest that 40% $O_{2}$ does not distort the fundamental metabolic identity of LNCaP spheroids, but rather optimally sustains and reinforces their characteristic, TCA‐driven energetic state [39, 40].

This oxygen‐supplementation strategy also expanded the measurable assay window. In our enzalutamide dose‐response assays, untreated spheroids maintained at 21% $O_{2}$ exhibited approximately 75% baseline viability, limiting the resolution of subsequent drug‐induced effects. In contrast, spheroids maintained at 40% $O_{2}$ retained near‐complete baseline viability and exhibited a more clearly resolved enzalutamide dose response (Figure 3l and Figure S8d). We therefore used 40% $O_{2}$ as a necrosis‐controlled 3D screening condition rather than as a model of tumor normoxia.

### 2.4 Experimental Screening Evaluates MAESTRO‐Prioritized Candidates and Pathway Enrichment

Using the optimized oxygen‐supplemented 3D culture system, we experimentally validated the synergistic potential of the 95 MAESTRO‐prioritized PKIs in combination with enzalutamide using the resistant 22Rv1 and MDVR cell lines. Following 72 h of co‐treatment with 1 μM PKI and 20 μM enzalutamide, we quantified cell viability (Figure 4a) and calculated Bliss synergy scores [41]. Consistent with standard conventions, “hits” were defined as compounds exhibiting a Bliss score >0.1 alongside a combinatorial efficacy ($E_{ab}$) superior to enzalutamide monotherapy [42, 43, 44]. This targeted screen identified 17 hits in 22Rv1 spheroids, yielding a 17.9% hit rate. Multiple inhibitors targeting the PI3K/AKT/mTOR signaling axis met the prespecified synergy criterion in both the 22Rv1 and MDVR spheroid models (Figure 4b and Figure S10a).

**FIGURE 4:**
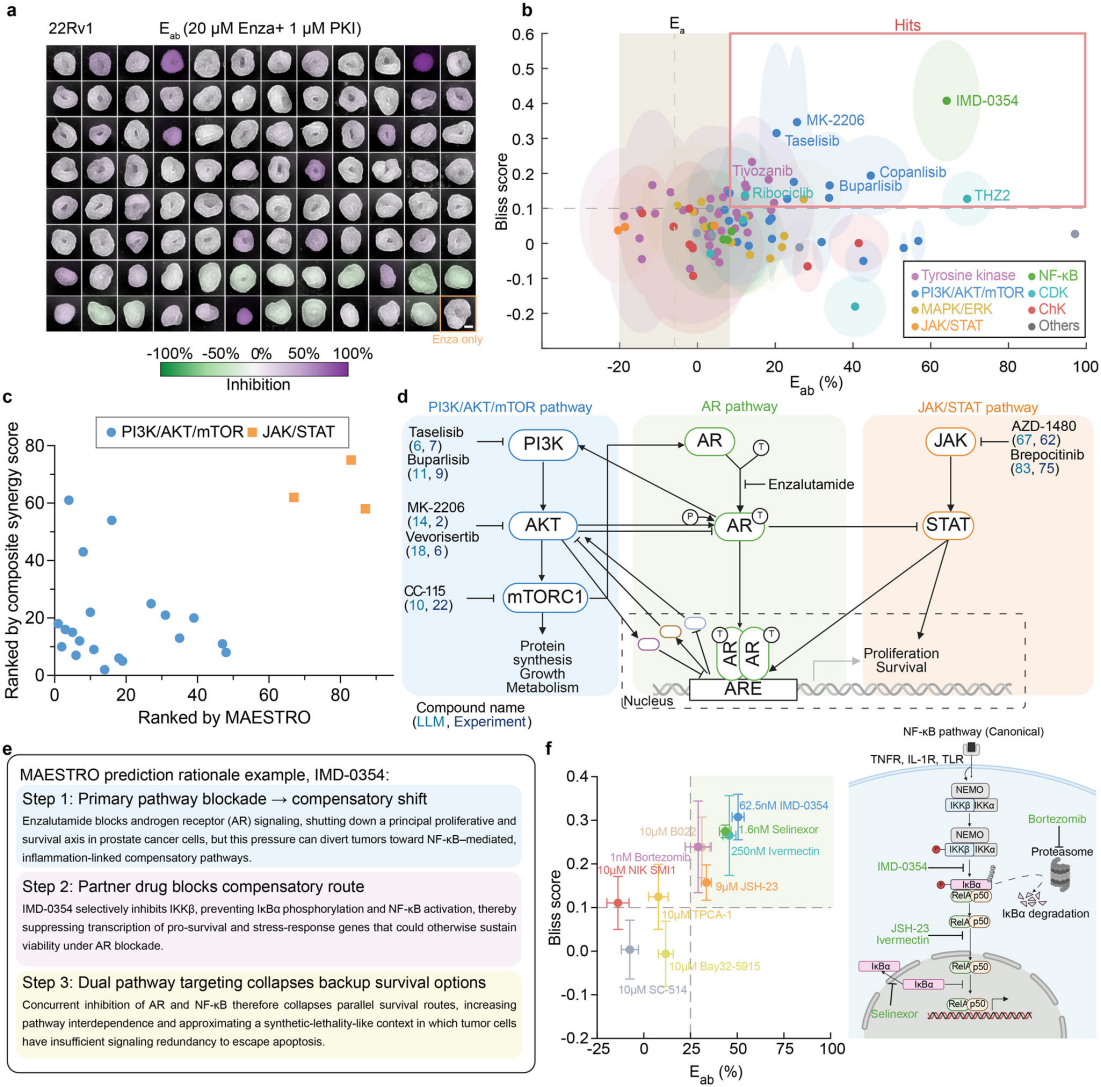
Experimental screening of MAESTRO‐prioritized kinase inhibitors in enzalutamide‐resistant models. (a) Representative pseudocolored bright‐field images of oxygen‐supplemented 3D 22Rv1 spheroids treated with 95 MAESTRO‐prioritized PKIs in combination with enzalutamide for 3 days under 40% $O_{2}$. Images are pseudocolored according to the combinatorial inhibition ratio ($E_{\text{ab}}$) relative to untreated controls. The enzalutamide monotherapy group (bottom left, orange box) serves as the therapeutic benchmark for synergy evaluation. Scale bar, 200 μm. (b) Scatter plot of $E_{\text{ab}}$ versus Bliss scores for the 95 PKIs tested in 22Rv1 spheroids. $E_{\text{ab}}$ was calculated as the inhibition rate (%) relative to the untreated group. Hits (red boxed region) are defined as compounds exhibiting $E_{\text{ab}}$ greater than enzalutamide monotherapy ($E_{a}$) and a Bliss score >0.1. Error ellipses denote the SD (n=4) for $E_{\text{ab}}$ (x‐axis) and Bliss scores (y‐axis). The vertical dashed line and shaded box represent the mean ± SD of the $E_{a}$. (c) Scatter plot comparing MAESTRO‐predicted rankings with composite synergy score rankings for PKIs targeting the PI3K/AKT/mTOR and JAK/STAT pathways. The composite synergy score was defined as ${\text{Bliss}}_{\mathrm{scaled}}\times E_{ab,\mathrm{scaled}}$, where mean Bliss and $E_{\text{ab}}$ (inhibition, %) were first averaged across 22Rv1 and MDVR spheroids for each PKI and then min–max scaled to 0–1 across all 95 screened PKIs. (d) Schematic illustration of signaling crosstalk between PI3K/AKT/mTOR and AR signaling compared to the JAK/STAT and AR signaling. Representative compounds are annotated with their MAESTRO predictions and experimental rankings derived from composite synergy scores. (e) MAESTRO‐generated pathway‐based rationale for the hit compound IMD‐0354, illustrating the model's step‐by‐step reasoning. (f) Pathway validation of the NF‐κB signaling axis as a therapeutic target. Left: Scatter plot of $E_{\text{ab}}$ versus Bliss scores for 10 NF‐κB inhibitors. $E_{\text{ab}}$ was calculated as the inhibition rate (%) relative to the untreated group (n=4). Right: Schematic of the canonical NF‐κB pathway, mapping the molecular targets of the identified hit compounds. Data are presented as mean ± SD.

Experimental rankings aggregated across both cell lines demonstrate strong concordance with MAESTRO's predicted drug prioritizations (Figure S10b,c). Notably, this experimental validation was restricted to the top 7.6% (95/1250 compounds) of MAESTRO‐prioritized candidates. By excluding the low‐ranking candidates, this subset has a truncated data distribution that inherently challenges the resolution of standard correlation metrics. Within this restricted set, PI3K/AKT/mTOR inhibitors were concentrated among experimentally synergistic combinations, whereas JAK/STAT inhibitors were largely nonresponsive (Figure 4c). This targeted stratification confirms that the framework's context‐aware reasoning effectively captures the underlying signaling architecture of prostate cancer. The model successfully prioritized the PI3K/AKT/mTOR axis, which is involved in reciprocal feedback with AR signaling and serves as a primary adaptive bypass mechanism [6, 7, 12]. Similarly, the model accurately deprioritized the JAK/STAT pathway due to its limited functional connectivity to the AR axis (Figure 4d). These findings indicate that MAESTRO's mechanism‐informed prioritization can isolate synergistic vulnerabilities from within massive unselected compound pools.

At the individual‐compound level, MAESTRO nominated the IKKβ inhibitor IMD‐0354 as a synergistic candidate. This prediction was experimentally confirmed across both enzalutamide‐resistant cell lines (Figure S10d). MAESTRO generated the pathway‐based hypothesis that articulated AR blockade forces a cellular reliance on inflammation‐mediated survival pathways, thereby creating a targetable dependency on compensatory NF‐κB signaling (Figure 4e). We subsequently investigated whether this predicted vulnerability represents a generalizable therapeutic axis by screening a targeted panel of ten structurally diverse NF‐κB inhibitors within the 22Rv1 spheroid system. This expanded validation yielded a 60% hit rate, with the majority of successful candidates predominantly antagonizing the canonical NF‐κB cascade (Figure 4f and Figure S10e). These findings collectively validate MAESTRO's generative hypothesis, establishing NF‐κB pathway inhibition as a robust, computationally prioritized strategy for overcoming enzalutamide resistance.

### 2.5 MAESTRO Deconvolves Multi‐Target Drug Effects and Identifies Berberine as a Synergistic Agent

After demonstrating MAESTRO's performance on selective, single‐target inhibitors, we next sought to investigate its ability to decipher the complex multi‐target interactions inherent to natural products (NPs). Although the target promiscuity of NPs traditionally impedes their development as precise monotherapies, this intrinsic polypharmacology renders them uniquely suited for combination regimens aimed at the multi‐nodal disruption of resistant signaling networks [45]. By deploying MAESTRO against a library of 3592 NPs, we interrogated the framework's ability to navigate these heterogeneous MoA profiles and prioritize candidate combinations across compounds with highly variable degrees of prior molecular annotation. Consistent with our kinase inhibitor screens, this NP prioritization recapitulated the signature “inverted U‐shaped” variance distribution (Figure 5a). This robust stratification facilitated the extraction of high‐confidence candidates whose pleiotropic MoA profiles functionally aligned with the orthogonal blockade of AR‐bypass pathways.

**FIGURE 5:**
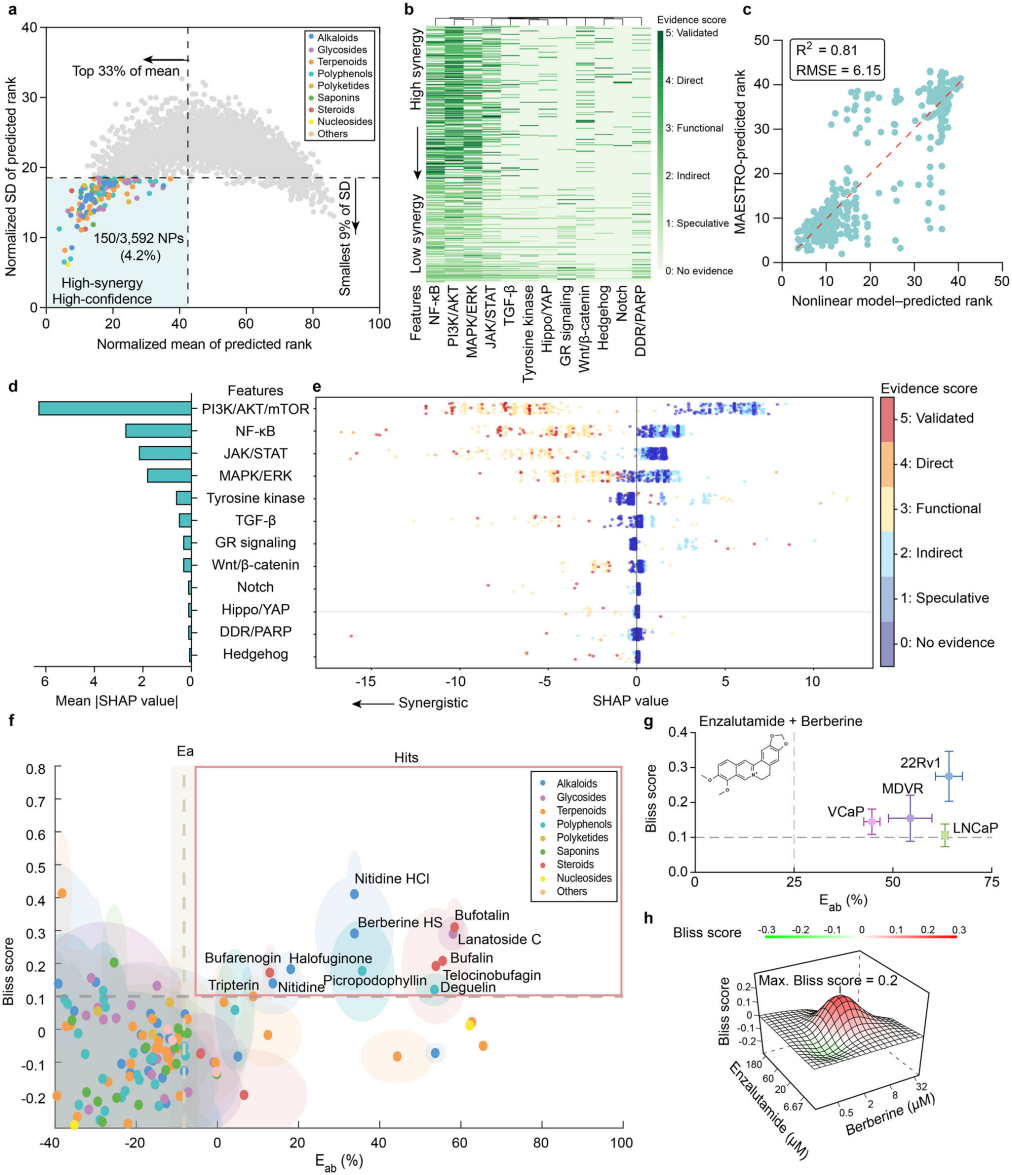
Deciphering MAESTRO's prioritization logic in polypharmacology and validating synergistic natural product candidates. (a) Distribution of 3592 natural products based on MAESTRO‐predicted mean synergy rank and rank SD (200 runs per compound). The highlighted region denotes 150 prioritized candidates for experimental validation, defined by a top 33% cutoff for mean rank and the lowest 9% cutoff for rank SD. (b) Heatmap displaying the evidence scores of 12 signaling features against the mean synergy rank for the top 500 compounds with the lowest rank SD. Evidence scores were quantified by the LLM, and the criteria are detailed in the Methods. Columns (features) were arranged by hierarchical clustering using correlation distance and average linkage, while rows (compounds) were ordered by ascending mean rank (top: high synergy; bottom: low synergy). (c) Correlation between Gradient Boosting Tree (GBT)‐predicted and MAESTRO‐predicted ranks for the 500 compounds with the lowest rank SD. GBT predictions were derived from 12‐dimensional evidence vectors. The red dashed line indicates the simple linear regression fit. $R^{2}$ denotes the coefficient of determination, and RMSE represents the root mean square error between the two ranking models. (d, e) Global feature importance (d) and feature impact distribution (e) derived from SHAP analysis. In (d), features are ranked by mean absolute SHAP value. In (e), points represent the distribution of SHAP values for 500 compounds across each of the 12 features, colored by evidence score, where negative values indicate a drive toward higher predicted synergy. (f) Experimental screening of 150 prioritized candidates (10μM) combined with Enzalutamide (20μM) in 22Rv1 spheroids. Scatter plot shows combination efficacy ($E_{\text{ab}}$) versus Bliss scores. $E_{\text{ab}}$ was calculated as the inhibition rate (%) relative to the untreated group. Hits (red box): $E_{\text{ab}}>$ enzalutamide monotherapy ($E_{a}$) and Bliss >0.1. Error ellipses: SD (n=4). Vertical line/shade: mean ± SD of $E_{a}$ (n=20). (g) Scatter plot of $E_{\text{ab}}$ versus Bliss score validating the synergy of berberine hydrogen sulphate and enzalutamide across four prostate cancer spheroid models (22Rv1, MDVR, VCaP, and LNCaP). Data points represent the most synergistic combination derived from the dose‐response matrix for each model. Dashed lines indicate the thresholds of $E_{\text{ab}}$ and Bliss scores for identifying hits. Data are presented as mean ± SD (n=8). (h) 3D synergy plot of the Berberine–Enzalutamide combination (n=3).

Moving beyond individual candidate rationales, we examined pathway‐level patterns in MAESTRO's prioritization across the natural‐product library. We quantified the evidentiary strength of each compound's MoA annotations across 12 canonical signaling pathways and evaluated how these pathway evidence scores were associated with MAESTRO‐derived ranks. Highly prioritized candidates were enriched for inhibitory annotations involving the NF‐κB, PI3K/AKT, MAPK/ERK, and JAK/STAT pathways (Figure 5b). To assess the extent to which pathway evidence scores could approximate these rankings, we trained a gradient‐boosted trees (GBT) surrogate model [46] that mapped MAESTRO‐derived ranks as a function of the pathway evidence scores (Figure S11a). The GBT surrogate achieved an $R^{2}$ of 0.81 across the 500 compounds in the low‐variance zone (Figure 5c), increasing to $R^{2}=0.93$ among the top 100 high‐confidence predictions (Figure S11b). These results indicate that pathway‐level annotations account for a substantial proportion of the variation in MAESTRO‐derived ranks. However, because the GBT is a separately trained surrogate, this analysis identifies annotation patterns associated with candidate prioritization but does not directly reveal the LLM's internal decision process or establish causal biological drivers.

To systematically decode the hierarchical signaling logic captured by the GBT surrogate model, we used SHAP (SHapley Additive exPlanations) analysis [47] to quantify the predictive contribution of individual pathway hubs and their interactions. This interpretability analysis identified PI3K/AKT/mTOR inhibition as the principal determinant of predicted synergy, followed by the NF‐κB, JAK/STAT, and MAPK/ERK pathways (Figure 5d). Notably, the accompanying SHAP swarm plot (Figure 5e) revealed pronounced dispersion among instances with high PI3K/AKT/mTOR evidence scores (red and orange dots), encompassing a broad distribution of SHAP values. This horizontal variance at a constant feature value indicates that PI3K/AKT/mTOR blockade does not operate as an isolated or additive variable. Rather, it engages in nonlinear interactions across the broader signaling landscape. Specifically, its synergistic impact could be heavily potentiated by the concurrent suppression of specific auxiliary pathways. Conversely, the SHAP values for distal networks such as the Notch and Hedgehog pathways tightly converged around zero irrespective of their evidence strength, demonstrating that these peripheral nodes exert negligible predictive leverage compared to the core survival cascades.

Applying the established High‐Confidence Zone (HCZ) criteria, defined by the lowest 9% rank variance and the top 33% mean rank, we isolated 150 lead candidates (4.2% of the total library) for experimental validation using the 3D 22Rv1 spheroid model. Recognizing the characteristically lower intrinsic potency of natural products relative to synthetic small molecules, we evaluated these candidates at dual concentrations (1 and 10 μM) against a constant 20 μM enzalutamide backbone. This targeted screen yielded a 15.3% hit rate, identifying 23 unique synergistic hits (17 hits at 10 μM and 12 hits at 1 μM, with 6 demonstrating cross‐dose activity). This finding confirmed MAESTRO's precision in prioritizing libraries (Figure 5f and Figure S12a). We then performed multi‐dose response surface modeling to narrow the leads down to four lead candidates: berberine, deguelin, nimbolide, and resibufogenin, based on the magnitude and reproducibility of their synergy scores (Figure S12b). We evaluated the generalizability of these leads via systematic cross‐validation across a broader panel of prostate cancer cell lines, encompassing both enzalutamide‐resistant (MDVR) and ‐sensitive (VCaP, LNCaP) phenotypes (Figure S12c– e). Berberine was selected for further characterization because it showed synergy with enzalutamide across all four models (Figure 5g). Furthermore, checkerboard assays in both ambient 2D (21% $O_{2}$) and oxygen‐supplemented 3D (40% $O_{2}$) 22Rv1 models (Figure 5h and Figure S13a) further demonstrated a broad region of positive Bliss interaction across multiple dose pairs within the tested in vitro concentration matrix.

To determine whether these validated synergies remained detectable under conventional 3D culture conditions, we re‐tested the four lead candidates in 22Rv1 spheroids cultured under ambient 21% $O_{2}$. The berberine–enzalutamide combination retained an attenuated synergistic signal, whereas the Bliss scores for deguelin, nimbolide, and resibufogenin fell below the prespecified synergy threshold (Figure S13b). Live/Dead assays revealed greater baseline core death under these conditions (Figure S13c), which reduced the assay's dynamic range and may have limited detection of additional treatment‐associated loss of viability. These findings indicate that oxygenation influences the sensitivity of 3D spheroid assays and that conventional culture conditions may underestimate some synergistic interactions.

### 2.6 Oxygenation‐Context Sensitivity Analysis Distinguishes the Screening Phenotype from Imposed Hypoxic and Hyperoxic States

We performed an oxygenation‐context sensitivity analysis to determine whether the experimental screening outcomes were more consistent with the original oxygen‐agnostic MAESTRO predictions or with predictions conditioned on severe hypoxic or sustained hyperoxic‐stress phenotypes. Two otherwise matched prompt contexts were constructed to encode the spheroid microenvironment, cellular state, and pathway dependencies expected under each oxygenation phenotype (Figure S14a). Rankings were generated for 1000 natural products, including the 150 experimentally tested candidates and 850 randomly sampled compounds.

Rankings generated under the hypoxia and hyperoxia contexts remained correlated with the baseline rankings (r = 0.953 and 0.847, respectively), indicating that oxygenation context modified rather than replaced MAESTRO's underlying prioritization hierarchy (Figure S14b). However, experimental hits were significantly deprioritized under both hypoxia and hyperoxia assumptions. Experimental non‐hits were significantly prioritized under the hypoxia context and showed no significant shift under the hyperoxia context (Figure S14c– d). Thus, imposing either extreme oxygenation phenotype reduced the correspondence between predicted rankings and the experimentally observed hits. These findings do not establish an absolute oxygen state within the spheroids, but they indicate that the drug‐response pattern obtained at 40% $O_{2}$ was not primarily reflective of the severe hypoxic or sustained hyperoxic‐stress phenotypes represented in the prompts.

### 2.7 Molecular and Transcriptomic Profiling Evaluates the MAESTRO‐Generated AMPK/mTORC1 Hypothesis

We next tested whether the cellular response was consistent with the AMPK/mTORC1 pathway hypothesis associated with berberine prioritization. We quantified the frequency of specific pathway nodes cited across 200 independent LLM prediction iterations, revealing that coordinated modulation of the PI3K/AKT/mTORC1 and AMPK signaling axes was implicated in the prioritization of berberine (Figure 6a). Our immunofluorescence staining results show that at 8 h, the combination treatment reduced the p‐S6/total S6 signal and increased p‐AMPKα/total AMPKα relative to vehicle, consistent with downstream mTORC1 modulation and AMPK activation, respectively (Figure 6b–c). To determine whether AMPK activation represented a transient response or persisted during treatment, we measured p‐AMPKα at 1, 8, and 48 h. Combination‐associated AMPK activation was detectable at 1 h, became more pronounced at 8 h, and remained elevated at 48 h (Figure S15a– b), showing elevated p‐AMPKα at the early, intermediate, and later sampled time points.

**FIGURE 6:**
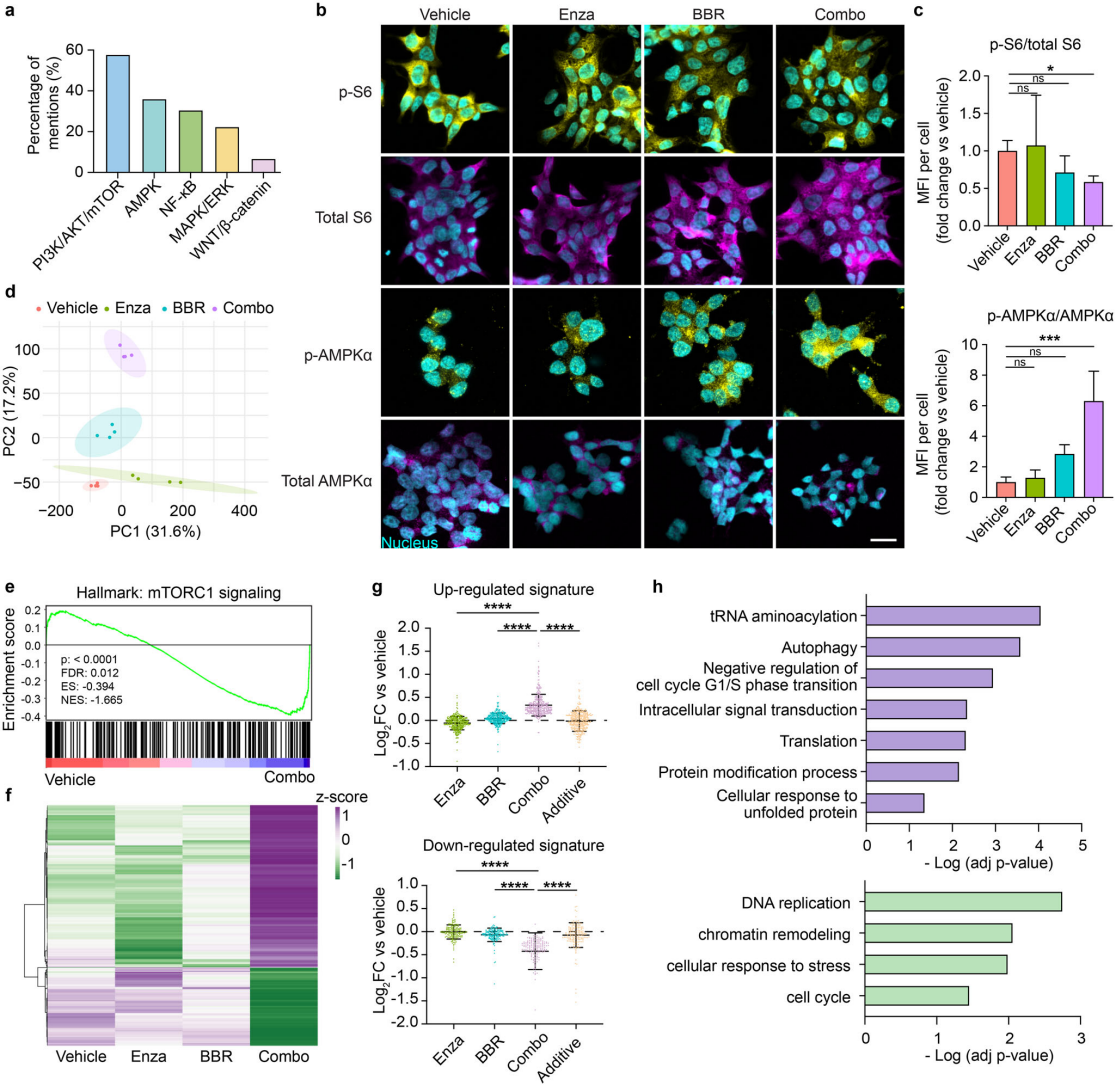
Experimental evaluation of the MAESTRO‐generated AMPK/mTORC1 pathway hypothesis for Berberine–Enzalutamide synergy. (a) Frequency analysis of MAESTRO‐generated pathway‐based rationales contributing to the synergy between berberine and enzalutamide. (b–c) Experimental validation of mTORC1 and AMPK pathway modulation. (b) Representative immunofluorescence images and (c) corresponding quantification of p‐S6/Total S6 (top) and p‐AMPKα/Total AMPKα (bottom) ratios in 22Rv1 cells treated with vehicle, 20μM enzalutamide (Enza), 5μM berberine (BBR), or the combination (Combo) for 8 h. Scale bar, 20 μm. Data in (c) are presented as the fold change of mean fluorescence intensity (MFI) per cell relative to vehicle (n=3, with an average of 613 cells analyzed per replicate). Statistical significance was determined by one‐way ANOVA followed by Dunnett's multiple comparisons test. (d) PCA plot visualizing transcriptomic variation ($\log_{2}$‐CPM) in 3D 22Rv1 spheroids across the four treatment groups after 8 h. Ellipses indicate 95% normal probability confidence regions (n=4). (e) GSEA plot demonstrating significant downregulation of the Hallmark mTORC1 signaling signature in the Combo group compared to the vehicle group. Normalized enrichment score (NES) and false discovery rate (FDR) are indicated. (f) Heatmap displaying the expression profiles of the “Combo Signature,” comprising genes synergistically upregulated (n=354) or downregulated (n=171) upon combination treatment. Rows (genes) were arranged by hierarchical clustering using correlation distance and average linkage. (g) Plot depicting expression changes ($\log_{2}\mathrm{FC}$) of the up‐regulated (top) and down‐regulated (bottom) signature genes across the treatment conditions relative to the vehicle, compared with the theoretical values for the additive effect of individual therapies. Statistical significance was determined by the Friedman test followed by post hoc pairwise Wilcoxon signed‐rank tests with Holm's correction for multiple comparisons. (h) Functional enrichment analysis of the upregulated (top) and downregulated (bottom) Combo Signature genes via g:Profiler. Data are presented as mean ± SD; * p<0.05, ** p<0.01, *** p<0.001, **** p<0.0001, ns: not significant.

We next examined whether AMPK activation functionally contributes to the berberine–enzalutamide interaction. We performed a complete 2×2×2 factorial experiment with the AMPK inhibitor BAY‐3827. Pharmacological inhibition of AMPK with BAY‐3827 reduced the Bliss score from 0.165 (95% bootstrap CI, 0.141–0.189) to 0.045 (95% CI, 0.024–0.067; ΔBliss =−0.120, 95% CI, −0.152 to −0.087; $p_{\text{boot}}<0.0001$), lowering the interaction below the prespecified synergy threshold of 0.1 (Figure S15c,d). This attenuation supports functional involvement of an AMPK‐associated, BAY‐3827‐sensitive component in the combination response, although the remaining positive Bliss score indicates that additional mechanisms may also participate. For the mTORC1 axis, the reduction in p‐S6 was accompanied by negative enrichment of the Hallmark mTORC1 signaling gene set after 8 h of treatment (Figure 6e). Together, these measurements support downstream mTORC1 modulation at the phosphoprotein and transcriptional levels. However, because upstream PI3K and AKT nodes were not examined dynamically, these data do not demonstrate sustained suppression of the complete PI3K/AKT/mTOR pathway.

Given the central role of AR signaling in the response to enzalutamide and the contribution of AR‐V7 to enzalutamide resistance, we examined whether the treatments altered AR‐FL and AR‐V7 protein abundance. We selected 22Rv1 for molecular profiling because it served as our primary screening model, exhibited robust combination synergy, and provided a stringent AR‐V7‐positive context in which to test whether the predicted metabolic‐survival vulnerability remained actionable. Immunoblotting showed that BBR alone and the combination reduced AR‐FL, with no significant difference between these treatments, whereas AR‐V7 remained unchanged (Figure S16a,b). Thus, the combination does not restore sensitivity by depleting AR‐V7 but remains effective despite its persistent expression. These findings support AMPK activation and mTORC1 modulation as a parallel survival‐axis vulnerability, with BBR‐mediated AR‐FL reduction representing a potentially contributing but unconfirmed mechanism. Consistent with this interpretation, synergy was also observed in models with different AR backgrounds and resistance mechanisms.

To characterize downstream responses associated with this pathway hypothesis, we performed bulk RNA sequencing on 3D 22Rv1 spheroids to systematically profile the global transcriptomic consequences of the combinatorial regimen. We focused on the 22Rv1 line, as it served as our foundational screening model and showed a reproducible combination interaction during empirical validation. PCA revealed that the combination induced a transcriptomic shift along the identical axis as the monotherapies, yet with an amplified magnitude of transcriptional perturbation (Figure 6d). This extended trajectory indicates a cooperative enhancement of downstream signaling rather than divergent pathway reprogramming. Furthermore, GSEA showed negative enrichment of the Hallmark mTORC1 signaling gene set, consistent with reduced mTORC1‐associated output (Figure 6e), corroborating our prior phosphoprotein immunostaining data.

We applied a subtractive transcriptomic filter to identify a combination‐associated transcriptional signature [48]. By systematically excluding transcripts significantly regulated by either single agent, we enriched for a differential expression signature uniquely driven by the combinatorial interaction. This filtering strategy identified 525 genes (354 up‐regulated and 171 down‐regulated) whose differential expression significantly surpassed the predicted additive effects of the constituent monotherapies (Figure 6f,g). Functional enrichment analysis demonstrated that the up‐regulated gene subset was enriched for adaptive stress responses, most notably autophagy, the unfolded protein response (UPR), and the negative regulation of cell cycle G1/S phase transition. These ontological profiles are consistent with AMPK‐associated stress responses, reduced mTORC1‐associated output, and decreased cell‐cycle activity [49, 50, 51, 52] (Figure 6h). Concurrently, the down‐regulated signature was predominantly enriched for DNA replication and cell cycle progression machinery, reflecting an abrogation of proliferative capacity. These transcriptomic changes were consistent with the MAESTRO‐generated pathway hypothesis, including AMPK‐associated stress responses, reduced mTORC1‐associated output, and decreased cell‐cycle activity. They do not, however, establish the complete causal mechanism of the combination response.

## 3 Discussion and Conclusion

Overcoming adaptive therapeutic resistance fundamentally depends on deciphering the complex interplay between core survival hubs and non‐linear signaling crosstalk. Identifying actionable synergistic vulnerabilities within these networks demands an analytical scalability that far exceeds the capacity of manual literature curation. MAESTRO addresses this challenge as a knowledge‐integration and prioritization framework that incorporates mechanistic augmentation [53], complementing correlational screening with mechanism‐informed prioritization. Here, mechanistic augmentation refers to conditioning the LLM on literature‐supported compound MoA annotations and disease‐relevant signaling relationships. Central to this approach is the translation of LLM rationales into quantifiable consensus rankings and rank‐variance metrics through ensemble sampling. By treating model stochasticity as a measurable source of predictive variation, we show that low rank variance identifies candidates for which predicted and experimental rankings are most concordant, while semantic analysis demonstrates alignment between the accompanying rationales and the supplied pathway context [54]. This variance‐gated “High‐Confidence Zone” methodology aligns with emerging approaches in high‐stakes biomedical AI [55, 56], in which generative consistency can serve as an empirically calibrated indicator of predictive reliability. By integrating disease‐relevant pathway context with compound MoA annotations, this platform provides a scalable strategy for prioritizing candidate combinations from large bioactive libraries while retaining interpretable, pathway‐based rationales.

Within this mechanism‐informed framework, pathway and intra‐pathway analyses showed that MAESTRO differentiated inhibitors targeting central signaling nodes from those targeting more peripheral effectors. This targeted prioritization of core signaling nodes aligns with established principles of oncogenic signaling, which posit that the effective abrogation of primary compensatory cascades is prerequisite to dismantling the reciprocal feedback loops sustaining tumor viability [6]. In the enzalutamide‐resistance context, this analysis prioritized concurrent modulation of the mTORC1 and AMPK axes as a candidate combination rationale. While the reciprocal crosstalk between these energetic and survival pathways is biologically documented [57], concurrent AMPK activation and attenuation of mTORC1‐associated output may represent an underexplored therapeutic strategy for overcoming enzalutamide resistance.

This pathway‐based rationale guided the experimental evaluation of berberine's polypharmacology [58]. By interpreting its pleiotropic profile, the model nominated berberine's capacity to re‐sensitize resistant phenotypes through AMPK activation together with downstream mTORC1 modulation. The detection of elevated p‐AMPKα at all three sampled time points and the marked reduction in Bliss synergy following BAY‐3827 treatment provide functional support for AMPK as a contributor to this interaction. In parallel, reduced p‐S6 and negative enrichment of the mTORC1 transcriptional program after 8 h are consistent with downstream mTORC1 modulation. These results should not, however, be interpreted as demonstrating sustained suppression of the complete PI3K/AKT/mTOR cascade, because the temporal responses of upstream PI3K and AKT nodes were not examined. Genetic or rescue experiments, direct perturbation of upstream PI3K/AKT signaling, and phosphoproteomic profiling, together with time‐resolved analysis of multiple pathway components, will be needed to define the feedback architecture connecting AMPK, mTORC1, and AR signaling during combination treatment.

Clinical translation of the berberine–enzalutamide combination will require strategies to increase berberine exposure, because conventional oral dosing produces low and variable systemic concentrations, with bioavailability below 1% [59, 60]. Two approaches warrant further evaluation. Hyaluronate‐based liposomes increased the relative oral bioavailability of berberine 4.38‐fold in rats [61], while dihydroberberine produced greater systemic exposure at a lower oral dose in a small randomized crossover human study [62]. Although neither study establishes prostate‐tissue exposure or the feasibility of the concentrations used here, they identify testable formulation and chemical‐modification strategies for subsequent pharmacokinetic and in vivo development.

Oxygen supplementation introduces an important trade‐off between preserving hypoxia‐associated tumor biology and maintaining a viable dynamic range for phenotypic screening. The 40% $O_{2}$ condition used here should not be interpreted as reproducing physiological tumor oxygenation or as being biologically inert. It reduced central cell death without detectable oxidative injury or disruption of the measured non‐core proliferation, apoptosis, epithelial, and lineage markers. The oxygenation‐context sensitivity analysis further showed that the observed drug‐response pattern aligned better with the oxygen‐agnostic baseline predictions than with predictions conditioned on severe hypoxia or sustained hyperoxic stress. Together, these findings support the condition as a necrosis‐controlled 3D screening model, not as a universal representation of prostate tumor oxygen physiology. Because hypoxia can create distinct therapeutic dependencies, combinations prioritized in this platform should ultimately be evaluated across complementary oxygenation conditions, and dedicated hypoxic models will be required when hypoxia‐dependent resistance is the biological question.

Cross‐model analyses showed concordant ranking behavior and predictive performance across proprietary and open‐weight LLMs, demonstrating that MAESTRO is not restricted to a specific proprietary architecture. Open‐weight models therefore provide a practical alternative to proprietary models for improving longitudinal reproducibility and reducing susceptibility to model drift and version updates. While MAESTRO demonstrates predictive utility, its current architectural constraints provide critical opportunities for systematic refinement. A primary limitation is the framework's reliance on text‐based biochemical annotations, which inherently confines its inferential scope to the boundaries of existing literature and may reduce accuracy for poorly characterized chemical entities. Evolving toward truly de novo molecular discovery will require the integration of structural descriptors, such as SMILES strings or graph‐based embeddings, into the model's reasoning context [63].

The present study is intentionally limited to early‐stage computational discovery and experimental evaluation in established 2D and 3D prostate cancer cell‐line models. Although the four‐model panel spans distinct AR states and enzalutamide sensitivities, it does not capture interpatient heterogeneity or establish clinical predictive validity. Moreover, the study does not establish in vivo efficacy, systemic safety, pharmacokinetics, or clinical dose feasibility. Translation will require validation in clinically annotated patient‐derived organoids or primary tumor cells, optimization of berberine exposure, direct tumor pharmacokinetic measurements, and efficacy and safety testing in an appropriate enzalutamide‐resistant animal model.

In conclusion, the MAESTRO platform demonstrates that the generative consensus of LLM outputs can be systematically harnessed to calibrate the reliability of pathway‐based hypothesis generation in drug discovery. By integrating mechanism‐informed prioritization with experimental evaluation, this study identified berberine–enzalutamide as an in vitro combination hit in enzalutamide‐resistant prostate cancer models. Beyond this specific application, MAESTRO offers a modular early‐discovery framework that could be adapted to other therapeutic contexts. Its immediate utility may lie in leveraging the extensive molecular annotations available for standard‐of‐care agents, such as chemotherapeutics or PARP inhibitors, to prioritize underexplored combination hypotheses. For emerging inhibitors with limited literature coverage, the framework could incorporate additional functional evidence, including preliminary screening data or CRISPR‐derived dependency maps, to strengthen the biological context used for prioritization. Together, the integration of mechanism‐informed computational prioritization with experimental screening provides a scalable workflow for identifying candidate combinations for subsequent biological and translational evaluation.

## 4 Materials and Methods

### 4.1 Architecture of the MAESTRO Framework

The MAESTRO pipeline constructs context‐augmented prompts by integrating drug‐MoA labels with disease‐specific signaling contexts. For drug‐MoA generation, we utilize PubTator3 [64] to retrieve biomedical articles, executing a Boolean search for each compound using the keywords: “mechanism,” “target,” “gene,” “protein,” “pathway,” “signaling,” “biological,” “disease,” “cancer,” “activate,” or “inhibit.” These articles are then processed by OpenAI GPT‐4o‐mini (temperature = 1.0) to synthesize concise profiles of molecular targets, downstream signaling, and potential AR signaling crosstalk. Simultaneously, the disease‐specific context is derived via a two‐stage RAG workflow from 13 curated review articles on prostate cancer resistance. A structured prompt explicitly queries 12 conceptual categories, including epigenetic modifiers, upstream/downstream regulators, and cellular phenotypes, to ensure a comprehensive signaling context that connects upstream regulators to downstream phenotypes.

To accommodate context window limitations and mitigate the performance degradation associated with excessive token loads, candidates are processed in batches of 50 compounds per prompt. We employ a balanced random sampling algorithm to ensure each candidate is evaluated with equivalent frequency against randomized cohorts, effectively mitigating batch‐specific bias. Ranking is performed using either proprietary or open‐weight LLMs, as specified for each analysis. Throughout this study, all LLM inference is performed with the temperature set to 1.0; all other decoding parameters are left at their default values unless otherwise specified. Inference with open‐weight LLMs is performed through the OpenRouter API (OpenRouter, Inc.), which provides a unified interface for accessing multiple LLM providers. Next, to quantify sampling variability and stabilize the output, we implement an ensemble consensus strategy where each candidate undergoes a minimum of 100 independent prioritizations (with up to 400 runs for specific analyses). Final consensus rankings and uncertainty metrics are derived from the distribution of these iterations.

The workflow is formally defined as follows (Figure 1a): let $D=\{d_{1},d_{2},\cdots ,d_{n}\}$ denote the library of candidate drugs. In each independent sampling iteration t, a subset $S_{t}\subset D$ is constructed via the balanced random sampling strategy. Specifically, at each iteration, MAESTRO first identifies candidates that have appeared in the fewest previous batches; candidates tied at that minimum count are sampled randomly until a 50‐compound batch is formed. Repeating this procedure ensures that every candidate is evaluated an equal or nearly equal number of times while continually changing its competitors, thereby reducing persistent cohort imbalance. Once each batch $S_{t}$ is constructed, the synergy assessment is modeled as a ranking function $R_{t}:S_{t}\to \left\{1,\cdots ,50\right\}$, which represents the reasoning process mapping a drug's context to a specific rank. Consequently, $R_{t}\left(i\right)$ denotes the rank assigned to drug $d_{i}$ in sampling run t. The final consensus prioritization is quantified by the mean rank $\mu_{i}=\frac{1}{T}\sum_{t=1}^{T}R_{t}\left(i\right)$, where T is the total number of iterations. The sampling variability is captured by the standard deviation $\sigma_{i}=\sqrt{VAR(R_{t}\left(i\right))}$.

### 4.2 Manual Factual Audit of LLM‐Generated MoA Annotations

Forty natural products were randomly sampled from the 3,592‐compound library. For each compound, MoA annotations were generated with and without RAG by GPT‐4o‐mini under otherwise matched conditions. The paired annotations were de‐identified, randomized, and evaluated without knowledge of the generation condition. Each annotation was decomposed into independently verifiable atomic MoA claims. Claims directly supported by published experimental evidence were scored as 1; claims with partial or insufficient support were scored as 0.5; and claims lacking support or contradicted by available evidence were scored as 0. The evidence‐supported rate for each annotation was calculated as the sum of claim scores divided by the total number of claims. Paired annotations were compared using a two‐tailed paired test.

### 4.3 Open‐Weight Model Evaluation and Generalizability Across Cancer Types

Core MAESTRO analyses were repeated using GPT‐OSS‐20B, GPT‐OSS‐120B [65], and Qwen3.5‐35B‐A3B [66] under the same prompt structure, sampling strategy, and rank‐aggregation procedure used for GPT‐4o. Experimental performance was evaluated using 150 AR inhibitor‐anchored drug pairs and 89 abiraterone‐anchored drug pairs tested in PC‐3 cells from DrugComb database. Cross‐backend concordance was further assessed using three independently sampled datasets, each containing 700 natural products. Spearman's ρ was used to compare mean ranks generated by each open‐weight model with GPT‐4o rankings. Recovery of the GPT‐4o‐defined top 5% was evaluated by AUROC, using the open‐weight model ranks as prediction scores.

Generalizability beyond prostate cancer was evaluated using two DrugComb datasets, each comprising 108 5‐fluorouracil–anchored combinations tested in HCT‐116 or SW‐620 cells. Zero‐shot predictions were generated using GPT‐OSS‐20B and the same MAESTRO ensemble and ranking workflow used for the prostate cancer benchmarks. Drug pairs were ordered by increasing rank SD, and Spearman's ρ between predicted and experimental rankings was calculated for progressively larger subsets, the complete dataset, and the HCZ, defined as the 25% of pairs with the lowest rank SD.

### 4.4 Benchmarking MAESTRO Against Deep Learning Synergy Prediction Models

Quantitative comparators were selected according to four criteria: (i) availability of a public implementation; (ii) compatibility with the drug and cell‐line features available for the benchmark; (iii) generation of numerical pair‐level outputs that could be converted to synergy rankings; and (iv) representation of complementary model architectures. MatchMaker was selected as a descriptor‐based regression model that integrates chemical and cell‐line gene‐expression features, whereas DeepDDS was selected as a graph‐based classification model that learns drug representations from molecular structures. DeepSynergy was not evaluated separately because it represents the same general descriptor‐ and gene‐expression‐based model class as MatchMaker. CancerGPT and SynerGPT were not included in the quantitative comparison because their required inputs, supervision, and prediction tasks differ from the present benchmark, and complete implementations and trained checkpoints were not publicly available.

The initial training pool for MatchMaker and DeepDDS comprised 226,717 drug combinations from DrugComb. Before training, we removed all records involving either abiraterone or the PC‐3 cell line. The abiraterone‐anchored combinations tested in PC‐3 cells, described above, were retained exclusively as the held‐out test set, thereby implementing a leave‐drug‐and‐cell‐out evaluation. Each deep learning model was trained and evaluated separately under Bliss‐ and Loewe‐based ground‐truth definitions. All other model settings were retained at the defaults specified in their public implementations.

MAESTRO was evaluated on the same held‐out dataset using GPT‐5‐mini and GPT‐OSS‐120B as the base LLMs. MAESTRO underwent no task‐specific training, fine‐tuning, or parameter updating and received no few‐shot demonstrations or experimental synergy labels; its outputs therefore represent zero‐shot predictions. For comparison across models, MAESTRO mean ensemble ranks, MatchMaker‐predicted synergy scores, and DeepDDS‐predicted synergy probabilities were each converted to predicted synergy ranks, with rank 1 denoting the strongest predicted synergy. Prediction performance was evaluated by comparing these ranks with the corresponding experimental rankings derived independently from Bliss or Loewe scores.

### 4.5 MAESTRO's Prediction Characterization

In our verification workflow, we cross‐referenced the 95 prioritized PKIs against published records to distinguish pre‐existing knowledge from novel inferences. Two independent researchers with biomedical domain expertise systematically searched Google Scholar for each candidate using the query string: “[Candidate Name] AND Enzalutamide”. Searches were performed between January 31 and February 8, 2025. We restricted the manual screening scope to the top 100 results sorted by relevance. Validated synergy reports were defined based on the following inclusion criteria: (i) both drug names were co‐present in the text; and (ii) the article explicitly reported a synergistic interaction or combination benefit in either in vitro or in vivo settings. To clarify what this verification procedure does and does not address, MAESTRO was supplied with compound‐level MoA annotations and disease‐specific signaling context, but not with the pair‐level synergy outcomes generated prospectively in this study. We therefore distinguish the intended use of prior biological knowledge from outcome leakage, defined here as access to the specific combination outcome used for evaluation. Because proprietary LLM providers do not disclose their complete pretraining corpora, latent exposure to all pair‐specific literature cannot be excluded. The literature cross‐validation consequently assessed whether the predicted combinations had previously been reported as synergistic under the prespecified criteria above, rather than whether the model had encountered the underlying pathway or MoA literature.

To evaluate the relationship between target engagement directness and MAESTRO's prioritization, we systematically processed 127 previously classified PI3K/AKT/mTOR inhibitors using the LLM alongside their MoA labels. We instructed the model to assign a “Target Involvement Score” (scale 1–10) based on the specific definitions provided in the prompt. The exact scoring criteria provided to the LLM were as follows: “PI3K–AKT–mTOR Target Involvement Score (1 to 10). This score measures how strongly a drug directly acts on the PI3K–AKT–mTOR signaling axis, based only on its known mechanism of action, target descriptions, and how it is classified in the literature. How to assign the score: Score 9–10: The drug is a core, high‐confidence inhibitor of PI3K, AKT, or mTOR. It is clearly described as an AKT inhibitor, PI3K inhibitor, mTOR inhibitor, or dual PI3K/mTOR inhibitor. These drugs are widely recognized and categorized in reviews as canonical PI3K‐axis inhibitors. Score 7–8: The drug strongly inhibits the PI3K pathway but not exclusively. It may target PI3K‐axis kinases along with other kinases, or inhibit a very close upstream regulator. PI3K/AKT/mTOR inhibition is still a major part of its pharmacology but shares space with other targets. Score 5–6: The drug is not labeled as a PI3K/AKT/mTOR inhibitor, but multiple studies show that it meaningfully suppresses this pathway under some conditions. These effects tend to be indirect or context‐dependent. Score 3–4: The drug has weak or occasional effects on the PI3K axis. It may reduce AKT phosphorylation only at high concentrations or in specific models, and this is not a major feature of its mechanism. Score 1–2: The drug has no meaningful involvement with the PI3K–AKT–mTOR axis. Its primary targets belong to unrelated biology (e.g., metabolic enzymes, immune receptors, malaria proteins, transcription modulators, Rac1/ROCK, GCN2, etc.).”

To perform a semantic analysis of the mechanism‐informed reasoning, we first encoded the LLM‐generated justifications (n=100 per compound) into high‐dimensional dense vectors using the all‐MiniLM‐L6‐v2 SentenceTransformer model [67]. We then projected these semantic embeddings into a two‐dimensional space to visualize latent clustering patterns using t‐SNE (perplexity: 30; initialization: PCA) and PCA. All visualizations were generated with a fixed random state of 40. For word cloud visualization, we stratified the 127 PI3K/AKT/mTOR inhibitors into three cohorts: Top (Rank 1–30), Middle (Rank 50–80), and Bottom (Rank 100–127). We aggregated the reasoning texts (n=100 per compound) within each cohort and removed standard English stopwords and domain‐specific generic terms (e.g., “inhibitor”, “kinase”, “signaling”). The WordCloud Python library was used to generate visualizations, scaling term size proportionally to frequency within the aggregated corpus.

To develop our LLM‐driven evidence scoring for MoA annotations, we established a 0–5 ordinal Evidence Score to correlate the degree of evidence supporting drug‐MoA labels with MAESTRO rankings. To minimize self‐validation bias, we employed Google Gemini 2.5 Flash as an independent evaluator, distinct from the annotating GPT‐4o‐mini, to analyze the natural product (NP) drug‐MoA labels. The model assigned scores across 12 key signaling pathways and molecular targets, yielding a 12‐dimensional feature vector based on the following strict criteria: “Score 0: No evidence. Criteria: No evidence that indicates any interaction; Score 1: Speculative (In Silico). Criteria: Computational prediction, molecular docking, or hypothesis only. No wet‐lab data; Score 2: Indirect (Associative). Criteria: Secondary effects (e.g., mRNA expression changes), correlations, or general stress responses without a clear mechanism; Score 3: Functional (In Vitro). Criteria: Demonstrated change in cellular signaling (e.g., Western blot showing reduced phosphorylation or nuclear translocation) in cell culture; Score 4: Direct (Biochemical). Criteria: Proof of physical binding or direct enzymatic inhibition (e.g., kinase assays, ligand displacement); Score 5: Validated (In Vivo/Clinical). Criteria: Confirmed mechanism of action driving efficacy in animal models (xenografts) or human clinical trials.”

To conduct the multivariate regression and SHAP interpretability analysis, we grouped the NP library into four subsets (top 100, top 500, top 1,000, and complete library) based on the lowest SD of MAESTRO‐predicted ranks. Independent multivariate regression analyses were performed for each subset using the Gradient Boosted Trees (GBT) algorithm [46] implemented in scikit‐learn. The GBT model utilized an ensemble of 300 decision trees with a learning rate of 0.05 and a maximum tree depth of 3 to balance complexity and generalization. A fixed random state was applied for reproducibility. We evaluated model performance by calculating the Coefficient of Determination ($R^{2}$) and Root Mean Square Error (RMSE) between MAESTRO‐predicted and GBT‐predicted ranks. Feature contributions were quantified using SHapley Additive exPlanations (SHAP) [47]. We computed local SHAP values using the TreeExplainer method (shap Python package) and determined global feature importance by calculating the mean absolute SHAP value for each evidence category.

### 4.6 Cell Culture and Generation of Oxygen‐Supplemented PCa Spheroids

LNCaP and 16D^CRPC^ cells were cultured in RPMI 1640 media (Gibco, Cat# 11875093) with 10% fetal bovine serum (FBS; Gibco, Cat# 26140079) and 1% penicillin/streptomycin (P/S; Gibco, Cat# 15140122). 22Rv1 cells were cultured in RPMI 1640 ATCC modification (Gibco, Cat# A1049101) with 10% FBS and 1% P/S. VCaP cells were cultured in DMEM (Gibco, Cat# 11995040) with 10% FBS and 1% P/S. C4‐2B MDVR cells [38] were received from Dr. Allen Gao, UC Davis, and cultured in RPMI 1640 media with 10% FBS, 1% P/S, and 20 μM enzalutamide (Apexbio, Cat# A3003). All 2D cells and 3D spheroids were maintained at 37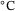, 5% ${\mathrm{CO}}_{2}$. Media changes were performed every 2–3 days, with subculturing conducted when cells reached 80–90% confluence. All cell lines tested negative for mycoplasma contamination by PCR and were used for experiments at low passage numbers.

To generate PCa spheroids with sizes of 500–600 μm, 22Rv1, C4‐2B MDVR, LNCaP, 16D^CRPC^, or VCaP cells were seeded at 10 000, 1000, 2000, 3000, and 15 000 cells per well, respectively, into 96‐well cell‐repellent V‐bottom plates (Greiner Bio‐One, Cat# 651970). Immediately after seeding, plates were centrifuged at 100 × g for 1 min to promote aggregation. Aggregates were cultured at 21% $O_{2}$ for 48 h to form compact spheroids. Each spheroid per well was then gently transferred to 96‐ or 384‐well ultra‐low‐attachment round‐bottom plates (Corning, Cat# 4515 and 4516, respectively) for optimal imaging quality. For oxygen‐level optimization assays, spheroids were cultured under 21%, 40%, 60%, and 95% $O_{2}$ for 72 h prior to downstream characterization using sealed sub‐chambers purged with pre‐mixed gas cylinders. Following the identification of 40% $O_{2}$ as the optimal condition, a tri‐gas incubator (Thermo Scientific) was employed to maintain 40% $O_{2}$ for all subsequent drug‐screening assays. In drug‐screening assays, PCa spheroids were treated with compounds and maintained at 40% $O_{2}$ for 72 h.

### 4.7 Immunofluorescence Staining

Following 72 h incubation at their respective oxygen levels, PCa spheroids were fixed in 4% paraformaldehyde (Thermo Scientific Chemicals, Cat# 043368‐9M), embedded in OCT compound, and snap‐frozen. Cryosections (12 μm) were prepared, then permeabilized and blocked in PBS containing 2% donkey serum and 0.25% Triton X‐100 (Sigma–Aldrich, Cat# X100) at room temperature for 30 min. After PBS washes, primary antibodies against Ki‐67 (Cell Signaling Technology, RRID: AB_2797703), cleaved caspase‐3 (CC3; Cell Signaling Technology, RRID: AB_2341188), E‐cadherin (Proteintech, RRID: AB_10697811), and γ‐H2AX (Thermo Fisher Scientific, RRID: AB_2573048) were applied in PBS with 0.125% Triton X‐100 and 0.5% bovine serum albumin (BSA; Fisher BioReagents, Cat# BP671‐10) and incubated at 4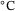 overnight. Primary antibodies were diluted according to the manufacturer's instructions. Following PBS washes, fluorophore‐conjugated secondary antibodies (1:1000) and 10 μg ${\mathrm{mL}}^{-1}$ Hoechst 33342 (Hoechst; Invitrogen Cat# H1399) were applied and incubated at room temperature for 1 h in the dark. Slides were mounted with ProLong Diamond Antifade Mountant (Invitrogen, Cat# 36970) and imaged on a Revolution fluorescence microscope (ECHO) using identical acquisition settings across groups. For quantitative analysis, image data were processed using QuPath software (v0.4.3) [68]. Cells were detected from the Hoechst channel using the Cell Detection tool (Gaussian sigma, 2.0 μm; minimum/maximum object area, 10 – 40 000 $\mu m^{2}$; cell expansion, 5.0 μm to define whole‐cell ROIs). Biomarker positivity was assigned using the Object Classifier based on nuclear mean intensity for Ki‐67 and γ‐H2AX, and expanded whole‐cell mean intensity for CC3. Positivity was reported as the ratio of marker‐positive cells to the total number analyzed, with identical detection parameters and thresholds applied across all groups.

For 2D monolayer cultures, 22Rv1 cells were seeded into 12‐well removable chamber slides (ibidi, Cat# 81201) at a density of 140 000 cells ${\mathrm{cm}}^{-2}$ and cultured for 24 h at 37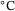 with 5% ${\mathrm{CO}}_{2}$. Cells were treated with vehicle, 20 μM enzalutamide (Enza), 5 μM berberine (BBR; MedChemExpress, Cat# HY‐N0716B), or the combination (Combo) for 8 h, unless otherwise indicated. Following treatment, cells were fixed in 4% paraformaldehyde containing phosphatase inhibitor cocktail (Cell Signaling Technology, Cat# 5870S) for 30 min on ice, then permeabilized and blocked in PBS containing 2% donkey serum and 0.25% Triton X‐100 at room temperature for 30 min. After PBS washes, primary antibodies against phospho‐S6 (p‐S6; Cell Signaling Technology, RRID: AB_10694233), total S6 (Cell Signaling Technology, RRID: AB_331355), phospho‐AMPKα (p‐AMPKα; Cell Signaling Technology, RRID: AB_2799368), and total AMPKα (Cell Signaling Technology, RRID: AB_10622186) were applied in PBS with 0.125% Triton X‐100 and 0.5% BSA and incubated at 4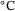 overnight. Primary antibodies were diluted according to the manufacturer's instructions. Following PBS washes, fluorophore‐conjugated secondary antibodies (1:1000) and 10 μg ${\mathrm{mL}}^{-1}$ Hoechst 33342 were applied and incubated at room temperature for 1 h in the dark. Slides were mounted with ProLong Diamond Antifade Mountant and imaged on a Zeiss Axio Observer Z1 (Carl Zeiss) inverted microscope equipped with an NL5+ line‐scanning confocal module (Confocal NL). Image acquisition was performed using Micro‐Manager software with identical settings across all groups. For quantitative analysis, image data were processed using a workflow integrating Cellpose and ImageJ. Nuclei were segmented from the Hoechst channel using Cellpose (model type, ‘nuclei') to obtain cell counts per image. Biomarker expression was quantified in ImageJ by generating above‐threshold masks for each channel. The integrated density was computed within the mask (mean intensity × mask area) and normalized by the nuclear count to yield mean fluorescence intensity per cell (MFI/cell). Identical segmentation parameters and intensity thresholds were applied across all treatment conditions.

### 4.8 Live/Dead Staining

Following 72 h incubation at their respective oxygen levels, PCa spheroids were washed twice with serum‐free medium and incubated with 2 μM Calcein‐AM (Biotium; live‐cell stain, Cat# 80011‐2), 5 μM propidium iodide (PI; Biotium; dead‐cell stain, Cat# 40017), and 10 μg ${\mathrm{mL}}^{-1}$ Hoechst 33342 at 37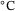 for 3 h. Spheroids were then imaged on a Revolution fluorescence microscope (ECHO) using identical acquisition settings across groups. For quantitative analysis, after setting a threshold to subtract the background, the Calcein‐AM and propidium iodide total intensity of the spheroids were measured in ImageJ and normalized to spheroid area.

### 4.9 Bulk RNA Sequencing and Transcriptome Data Analysis

To characterize the transcriptomic profiles of LNCaP cells under different culture conditions, cells were seeded as 2D monolayers (5000 cells ${\mathrm{cm}}^{-2}$) or 3D spheroids (3000 cells per well) and cultured at 21% $O_{2}$ for 3 days. Subsequently, cultures were maintained under 21% $O_{2}$ or 40% $O_{2}$ for an additional 5 days. Total RNA was extracted and purified by Direct‐zol RNA Miniprep Plus Kits (Zymo Research, Cat# R2072) according to the manufacturer's instructions. The extracted RNA samples were sent to Novogene Corporation Inc. (Sacramento, CA) for quality control, library construction, and sequencing. Briefly, RNA degradation and contamination were monitored on 1% agarose gels. RNA purity was checked using the NanoPhotometer spectrophotometer (IMPLEN, CA, USA). RNA integrity and concentration were quantified using the RNA Nano 6000 Assay Kit of the Bioanalyzer 2100 system (Agilent Technologies). High‐quality RNA samples that passed QC were used to construct sequencing libraries using the NEBNext Ultra RNA Library Prep Kit for Illumina (NEB, USA) with poly‐T oligo‐attached magnetic beads for poly‐A enrichment. The libraries were sequenced on an Illumina NovaSeq 6000 platform to generate 150‐bp paired‐end reads at a depth of 20 million reads per sample.

For data analysis, raw reads were processed using FastQC and Trim Galore to remove adapters, low‐quality bases (Q<25), and the first 10 bp of the 5’ end to eliminate potential bias. The cleaned reads were aligned to the human reference genome, GRCh38, using STAR, and gene counts were normalized to counts per million (CPM) and analyzed for differential expression using the limma R package. Gene Set Enrichment Analysis (GSEA) was performed using the WebGestalt (WEB‐based GEne SeT AnaLysis Toolkit) [69] web server. Genes were ranked by their t‐statistics derived from the limma analysis, and enrichment was assessed against the Reactome pathway database. Pathways with a False Discovery Rate (FDR) <0.05 were considered significantly enriched.

To investigate the transcriptional changes associated with BBR‐Enza synergy, compact 22Rv1 spheroids (48 h post‐seeding) were then treated with vehicle, 20 μM Enza, 5 μM BBR, or the combination under 40% $O_{2}$ for 8 h. Total RNA was extracted and purified by Direct‐zol RNA Miniprep Plus Kits according to the manufacturer's instructions. The extracted RNA samples were submitted to the UCLA Technology Center for Genomics & Bioinformatics (TCGB). Prior to library preparation, RNA integrity was assessed using an Agilent TapeStation system, with all samples exhibiting an RNA Integrity Number >7. Sequencing libraries were generated using the ABclonal FAST mRNA Library Prep Kit with poly‐A selection. The libraries were sequenced on an Illumina NovaSeq X Plus platform to generate 50‐bp paired‐end reads at a depth of 35 million reads per sample. For data analysis, sequencing data quality was confirmed with >90% of bases achieving a Q30 score. High‐quality reads were aligned to GRCh38 using STAR. Gene counts were normalized to CPM and analyzed for differential expression using the limma R package. GSEA was performed using the GSEA software (Broad Institute). Genes were ranked using the log2 ratio of classes metric based on normalized counts (CPM), and enrichment was assessed against the Hallmark gene set collection (MSigDB) using 2000 gene set permutations. Pathways with a nominal p‐value <0.05 and an FDR <0.05 were considered significantly enriched.

### 4.10 Combo Signature Gene Set Identification and Function Enrichment Analysis

To identify a transcriptomic signature specific to the synergistic interaction between BBR and Enza, we employed a subtractive filtering strategy adapted from Fuertes et al. [48]. This refined approach was designed to rigorously remove monotherapy‐driven gene expression changes and retain only those genes uniquely associated with the combination response. For all comparisons, statistical significance was defined as an adjusted p‐value <0.05, and genes that met this threshold were identified as differentially expressed genes (DEGs). To exclude the independent effects of BBR (List 1), we first selected DEGs from the “Combination vs. Enza” comparison (5,662 genes). From this list, we excluded genes that were significantly changed in the same direction (based on the sign of log2 fold change) in the “BBR vs. Control” comparison. Genes that were either non‐significant or significantly regulated in the opposite direction in the BBR monotherapy were retained to capture newly induced effects or expression reversal, resulting in 5258 genes. Similarly, to exclude the independent effects of Enza (List 2), we selected DEGs from the “Combination vs. BBR” comparison (1,667 genes) and excluded genes that were significantly changed in the same direction in the “Enza vs. Control” comparison, retaining those that were non‐significant or significantly regulated in the opposite direction under Enza monotherapy (673 genes). Finally, the intersection of List 1 and List 2 yielded 525 genes, comprising 354 up‐regulated and 171 down‐regulated genes, which were designated as the “Combo Signature.”

To verify the synergistic expression patterns of the identified “Combo Signature”, the expression fold changes of the Combo Signature genes were compared against the monotherapies and their expected additive effect, which was defined using the formula: ${\text{Log2FC}}_{\text{Additive}}={\text{Log2FC}}_{\text{Enza}}+{\text{Log2FC}}_{\text{BBR}}$. Statistical differences across these four conditions were assessed using a Friedman test, followed by post‐hoc pairwise comparisons using paired Wilcoxon signed‐rank tests with Holm correction. Subsequently, to elucidate the biological functions of the signature, the 354 up‐regulated and 171 down‐regulated genes were analyzed separately using g:Profiler [70]. Enrichment was assessed against the Gene Ontology Biological Process (GO:BP) database using the g:SCS significance threshold of <0.05, and representative enriched pathways are presented in Figure 6h.

### 4.11 ‐Glucose Isotope Tracing and Metabolomic Analysis

Formed LNCaP spheroids (48 h post‐seeding) were cultured under 21% or 40% $O_{2}$ for 72 h. To trace glucose metabolism, the complete culture medium was replaced with glucose‐free RPMI medium (Gibco, Cat# 11879020) containing 10% dialyzed FBS (Gibco, Cat# A3382001) and 2 g $L^{-1}$ uniformly labeled D‐[U‐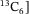 glucose (Cambridge Isotope Laboratories, Cat# CLM‐1396‐1) for the final 24 h of the incubation period. For each condition, 64 spheroids were collected and washed with ice‐cold 150 mM ammonium acetate buffer. Intracellular metabolites were extracted with ice‐cold 80% methanol containing 0.1 mM norvaline (Sigma–Aldrich, Cat# N7502‐25G) as an internal standard. Spheroids were homogenized by brief vortexing, followed by incubation at $-80^{\circ }C$ for 30 min to facilitate extraction. Lysates were centrifuged at 16 000 × g for 5 min at 4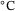, and supernatants were collected and dried under nitrogen using an EZ‐2 Elite centrifugal evaporator (Genevac). Cell pellets were collected, and genomic DNA was purified using the GeneJET Genomic DNA Purification Kit (Thermo Scientific, Cat# K0721). Subsequently, DNA concentration was quantified using a NanoDrop 2000C spectrophotometer (Thermo Scientific). Total DNA content was calculated and used to normalize metabolite abundances for downstream comparative analyses.

Dried metabolite extracts were sent to the UCLA Metabolomics Center for LC–MS analysis. Briefly, dried metabolites were reconstituted in 100 μL of 50% acetonitrile (ACN) in water, and 5 μL was loaded onto a Luna ${\mathrm{NH}}_{2}$ column (3 μm, 100 Å, 150 × 2.0 mm; Phenomenex). Chromatographic separation was performed on a Vanquish Flex UHPLC system (Thermo Scientific) using mobile phase A (5 mM ammonium acetate, pH 9.9) and mobile phase B (ACN) at a flow rate of 200 μL $\min^{-1}$. A linear gradient from 15% A to 95% A over 18 min was followed by 7 min isocratic flow at 95% A and re‐equilibration to 15% A. Metabolites were detected on a Q Exactive Orbitrap mass spectrometer (Thermo Scientific) operated in polarity‐switching, full‐scan mode over an m/z range of 70–975 with a resolution of 70 000. MAVEN (v8.1.27.11) was used to quantify targeted metabolites by peak area (AreaTop), based on accurate mass measurements (<5 ppm) and previously verified retention times. 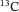 natural abundance correction was performed using AccuCor. Relative amounts of metabolites were calculated by summing up the intensities of all detected isotopologues of a given metabolite. Downstream data processing and statistical analyses were performed using in‐house R scripts.

### 4.12 PSA Measurement

Following 72 h incubation at their respective oxygen levels, conditioned media were collected from LNCaP spheroids. PSA concentrations were quantified using the Human Kallikrein 3/PSA Quantikine ELISA Kit (R&D Systems, Cat# DKK300) according to the manufacturer's instructions. DNA from spheroids was extracted with the GeneJET Genomic DNA Purification Kit and quantified on a NanoDrop 2000C spectrophotometer. PSA values were then normalized to total DNA content per replicate.

### 4.13 Intracellular Reactive Oxidative Species (ROS) Measurement

Following 72 h incubation at their respective oxygen levels, LNCaP spheroids were washed with PBS and incubated with 10 μM $H_{2}\mathrm{DCFDA}$ (Invitrogen, Cat# D399), a fluorescent probe for intracellular ROS, in PBS at 37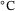 for 1 h under their respective oxygen conditions. Following incubation, spheroids were washed twice with serum‐free medium, and fluorescence intensity (Ex/Em: 495/528 nm) was quantified using a Synergy H1 microplate reader (BioTek Instruments). ROS levels were normalized to the total DNA content of each replicate.

### 4.14 Dose‐Response Analysis, Small‐Molecule Screening, and Cell‐Viability Assays

For Enza dose–response assays, PCa spheroids (48 h post‐seeding) were treated with Enza at concentrations ranging from 512 to 2 μM (twofold serial dilutions). Vehicle controls were treated with matched concentrations of DMSO. The spheroids were incubated under 21% or 40% $O_{2}$ for 72 h. Absolute cell viability was quantified by live/dead staining and expressed as the ratio of live cells to total cells (live ÷ [live + dead]). Half‐maximal inhibitory concentration (${\mathrm{IC}}_{50}$) values were determined by fitting dose‐response data to a four‐parameter logistic non‐linear regression model using GraphPad Prism software.

Small‐molecule screening and validation were performed in a tiered approach. (1) Primary screen: 22Rv1 spheroids were treated with 95 MAESTRO‐prioritized PKIs (Molecular Screening Shared Resource, UCLA) or 150 NPs (TargetMol) at fixed concentrations (1 or 10 μM), either as single agents or in combination with 20 μM Enza. (2) Multi‐dose response validation: The top 14 NP hits were re‐evaluated in 22Rv1 spheroids at 1–20 μM in the presence of 20 μM Enza. (3) Cross‐model validation: Four lead candidates (berberine hydrogen sulphate, nimbolide, deguelin, and resibufogenin) were tested in MDVR, LNCaP, and VCaP spheroids (1–20 μM NPs) combined with model‐specific Enza concentrations (20 μM for MDVR, 10 μM for LNCaP, and 20 nM for VCaP). (4) Synergy matrix: A full checkerboard validation was performed for berberine hydrogen sulphate in oxygen‐supplemented 22Rv1 spheroids, assessing combinations of berberine hydrogen sulphate (0.5–32 μM) and Enza (6.67–180 μM). Across all stages, unless otherwise specified, spheroids (48 h post‐seeding) were incubated with drugs under 40% $O_{2}$ for 72 h. Cell viability was quantified using the CellTiter‐Glo 3D assay (Promega, Cat# G9683) and normalized to untreated controls.

### 4.15 Synergy Score Identification

Synergy was evaluated using the Bliss independence model [41]. Inhibition ratio (E, %) was calculated as: E=1−cell viability(%). Bliss synergy scores were then computed using: $E_{ab}-\left(E_{a}+E_{b}-E_{a}\times E_{b}\right)$, where $E_{a}$ and $E_{b}$ are the inhibition ratios for the monotherapy, and $E_{ab}$ is the inhibition ratio for the combination therapy. Compounds with $E_{ab}$ greater than $E_{\text{Enza}}$ and a Bliss score >0.1 were designated as hits and prioritized for subsequent multi‐dose validation. The Bliss scores for the full dose–response matrix of the BBR–Enza combination were computed in SynergyFinder [71] and visualized as a 3D surface plot.

### 4.16 Effect of AMPK Inhibition on Enza–BBR Synergy

To evaluate the involvement of AMPK signaling in the interaction between Enza and BBR, the AMPK inhibitor BAY‐3827 (BAY; MedChemExpress, Cat. No. HY‐112083) was incorporated into the combination assay. 22Rv1 cells were seeded in 96‐well plates at 10 000 cells per well and allowed to adhere for 24 h. Cells were then treated for 72 h using a complete 2×2×2 factorial design comprising enzalutamide (Enza; 20 μM), berberine (BBR; 5 μM), and BAY (0.5 μM). Cell viability was quantified using the alamarBlue HS assay (Invitrogen, Cat. No. A50101) and normalized to vehicle. The BAY‐conditioned enzalutamide–berberine Bliss score was calculated using the BAY‐only condition as a reference:

$$\begin{array}{ccc} {\mathrm{Bliss}}_{(\mathrm{Enza}+\mathrm{BBR})|\mathrm{BAY}} & = & I_{(\mathrm{Enza}+\mathrm{BBR})|\mathrm{BAY}}\\ & & -\;\left[I_{\mathrm{Enza}|\mathrm{BAY}}+I_{\mathrm{BBR}|\mathrm{BAY}}-I_{\mathrm{Enza}|\mathrm{BAY}}\times I_{\mathrm{BBR}|\mathrm{BAY}}\right], \end{array}$$

where $I_{X|\mathrm{BAY}}$ denotes the inhibition produced by treatment X in the presence of BAY relative to the BAY‐only condition. The terms $I_{\mathrm{Enza}|\mathrm{BAY}}$, $I_{\mathrm{BBR}|\mathrm{BAY}}$, and $I_{(\mathrm{Enza}+\mathrm{BBR})|\mathrm{BAY}}$ correspond to the Enza + BAY, BBR + BAY, and Enza + BBR + BAY treatment groups, respectively. The change in Bliss score was calculated as the BAY‐conditioned score minus the corresponding unconditioned score. Percentile‐based 95% bootstrap confidence intervals for the Bliss scores were estimated using 20 000 resamples. Statistical significance of ΔBliss was assessed using a two‐sided, null‐centered bootstrap test against zero based on the same 20 000 resamples.

### 4.17 Western Blot Analysis

22Rv1 cells were seeded at 80 000 cells per well and allowed to adhere for 24 h. Cells were subsequently treated for 24 h with vehicle, 20 μM Enza, 5 μM BBR, or their combination. Vehicle control cells received equivalent volumes of DMSO and $H_{2}O$. Following treatment, cells were collected, washed twice with PBS, and pelleted by centrifugation at 300 × g for 3 min. Cell pellets were lysed in RIPA buffer supplemented with protease inhibitor (1:100) and universal nuclease (1:10 000). Lysates were clarified by centrifugation at 17 000 × g for 15 min at 4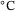, and the supernatants were collected. Protein concentrations were determined using a BCA assay. Equal amounts of protein were mixed with 1× SDS sample buffer, heated at 100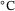 for 5 min, separated by SDS‐PAGE, and transferred onto nitrocellulose membranes.

Membranes were blocked with 5% non‐fat milk for 1 h and incubated overnight at 4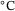 with antibodies against AR (Santa Cruz Biotechnology, RRID: AB_626671) and GAPDH (Santa Cruz Biotechnology, RRID: AB_627678), followed by incubation with an HRP‐conjugated goat anti‐mouse IgG (H+L) secondary antibody (Thermo Fisher Scientific, RRID: AB_228302) for 1 h at room temperature. Protein bands were detected using Pierce ECL Western Blotting Substrate (Thermo Fisher Scientific, Cat# 32106). Bands corresponding to AR‐FL and AR‐V7 were identified based on their expected molecular masses. Band intensities were quantified using ImageJ as previously described [72]. The abundances of AR‐FL and AR‐V7 were separately normalized to the corresponding GAPDH loading control and expressed as fold changes relative to the vehicle control.

### 4.18 Evaluation of Oxygenation‐Context Sensitivity in MAESTRO Predictions

To assess the sensitivity of MAESTRO prioritization to physiological assumptions regarding oxygenation, a dataset of 1000 natural products (NPs), comprising 150 experimentally screened candidates and 850 randomly sampled compounds from the remaining library, was evaluated under normoxia, hypoxia, and hyperoxia phenotypes. Predictions for the normoxia phenotype (i.e., oxygen‐agnostic baseline) were generated with no additional oxygen‐specific information, whereas predictions for the hypoxia and hyperoxia phenotypes incorporated the indicated physiological assumptions regarding the spheroid oxygen microenvironment, cellular state, and pathway dependencies. The complete context prompts are provided in the Supporting Text. All three conditions were evaluated using GPT‐OSS‐20B with identical MoA annotations, ranking instructions, and inference settings. Compounds were globally ranked according to their mean ranks. ΔRank was calculated by subtracting the normoxia‐predicted rank from the corresponding hypoxia‐ or hyperoxia‐predicted rank, with negative and positive values indicating prioritization and deprioritization, respectively. For this analysis, among the 150 experimentally screened compounds, those with experimental Bliss scores >0.10 were classified as hits (n=42), whereas those with Bliss scores <−0.10 were classified as non‐hits (n=15). For each group, the 95% confidence interval for the mean ΔRank was estimated using 20 000 compound‐level bootstrap resamples. Statistical significance relative to zero was assessed using two‐sided null‐centered bootstrap tests, with Benjamini–Hochberg correction applied across the four prespecified comparisons.

### 4.19 Statistical Analysis

Unless otherwise stated, quantitative data are presented as mean ± standard deviation (SD), with n denoting biologically independent replicates as indicated in the figure legends. Statistical analyses were performed using GraphPad Prism (v9.0; GraphPad Software) or R (v4.3.0). For two‐group comparisons, statistical significance was determined using unpaired two‐tailed Student's *t*‐tests. For comparisons involving more than two groups, one‐way Analysis of Variance (ANOVA) was performed, followed by Dunnett's post‐hoc tests for multiple comparisons. To verify the synergistic expression patterns of the identified “Combo Signature,” the Friedman test followed by paired Wilcoxon signed‐rank tests with Holm correction was utilized. For RNA‐seq differential expression, statistical testing and multiple‐testing correction were performed as described in the corresponding section. Unless otherwise stated, a p‐value <0.05 was considered statistically significant.

## Author Contributions

C.H.L., A.S.G., and N.Y.C.L. conceived the project; C.H.L., L.E., Y.H., C.J.H., and N.Y.C.L. developed the computational framework; C.H.L., K.S., L.K., A.B., J.D., Z.C., and H.H. conducted the experiments; C.H.L., K.S., L.K., A.B., J.D., Z.C., H.H., W.Y., A.L., R.D., T.S., A.S.G., and N.Y.C.L. analyzed the results. All authors reviewed the manuscript.

## Ethics Statements

This study does not involve a clinical trial as defined by the International Committee of Medical Journal Editors (ICMJE) or the WHO, and therefore clinical trial registration was not required. No written consent has been obtained from the patients as there is no patient identifiable data included in this study.

## Conflicts of Interest

The authors declare no conflicts of interest.

## Supporting information


**Supporting File**: advs78186‐sup‐0001‐SuppMat.pdf.

## Data Availability

The raw and processed RNA sequencing data generated in this study have been deposited in the Gene Expression Omnibus (GEO) database under accession codes [GSE317709] and [GSE317905]. The drug screening datasets generated and analyzed during the current study, including the MAESTRO‐predicted rankings, sampling variability metrics, and experimental Bliss synergy scores for the 1250 protein kinase inhibitors and 3592 natural products, are provided in the Supplementary Data files. Publicly available datasets used for benchmarking and knowledge‐augmentation were accessed from the DrugComb database (v1.4) at https://drugcomb.fimm.fi and PubTator3 at https://www.ncbi.nlm.nih.gov/research/pubtator3/. Any other data supporting the findings of this study are available from the corresponding authors upon reasonable request.
