# Supplementary material for: Mechanism‐Informed Language Modeling and Oxygenated 3D Screening Identify Berberine–Enzalutamide Synergy in Prostate Cancer Models
Source: Adv Sci (Weinh). 2026 Oct 8:e78186. Online ahead of print. doi: 10.1002/advs.78186 (PMC13650049; doi:10.1002/advs.78186)
Supplement: Supplementary file 1 [file ADVS-9999-e78186-s001.pdf]

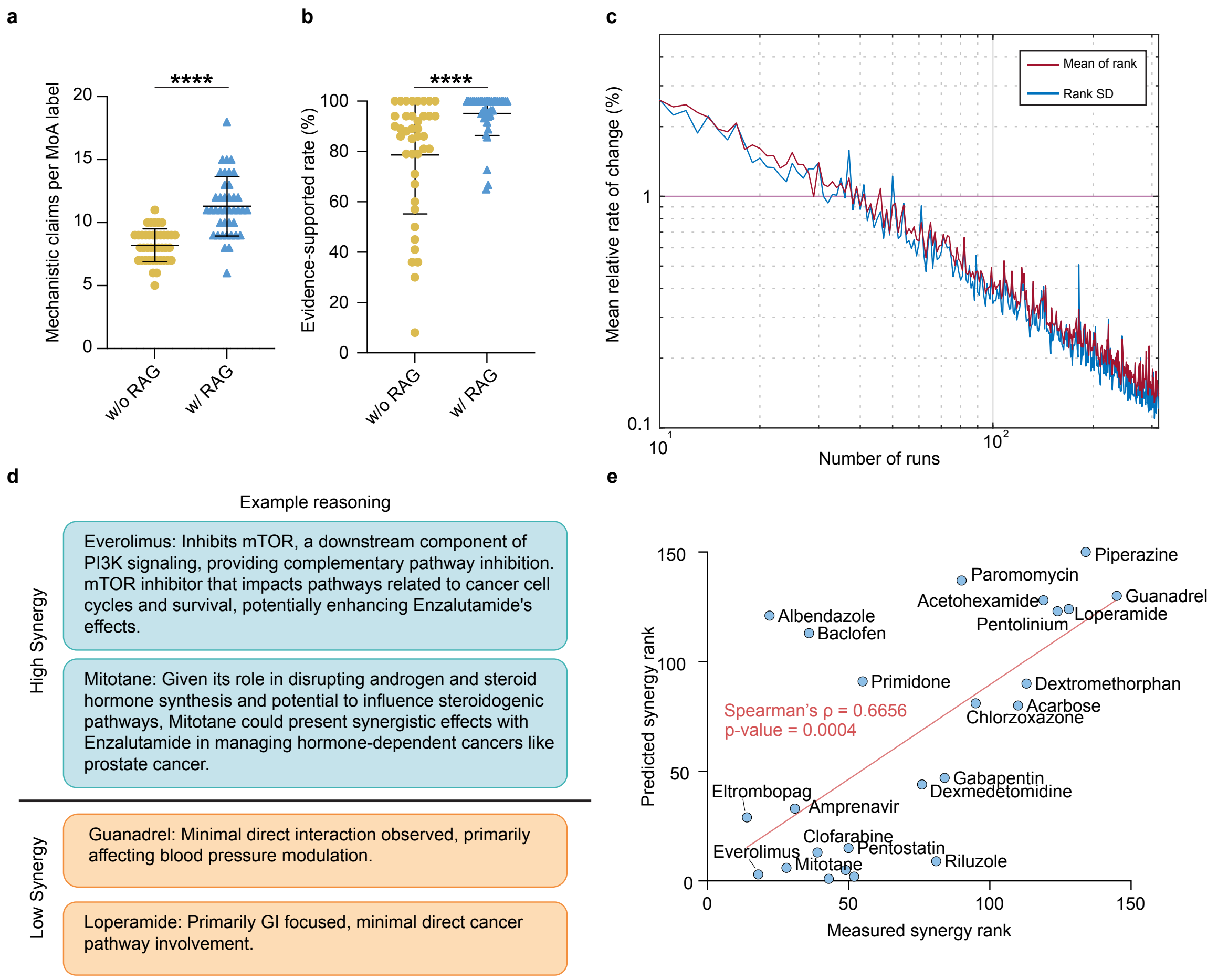

Supplementary Figure 1. RAG increases evidentiary support for generated MoA annotations, while repeated sampling stabilizes MAESTRO rankings. (**a–b**) Number of atomic mechanistic claims per annotation (**a**) and corresponding evidence-supported rates (**b**) for MoA annotations generated with or without RAG for 40 randomly selected natural products. Deidentified annotations were decomposed into independently verifiable claims and evaluated against published experimental evidence. Claims were scored as supported (1), partially supported (0.5), or unsupported or contradicted (0). Evidence-supported rates were calculated as the sum of claim scores divided by the total number of claims. Lines indicate mean ± SD; **** $p < 0.0001$ by two-tailed paired test. (**c**) Plot of mean relative rate of change (%) versus the number of runs per compound. (**d**) Representative examples of MAESTRO-generated rationales distinguishing between high-synergy and low-synergy predictions. **(e)** Predicted synergy ranking versus experimentally measured synergy ranking for the 24 representative drugs exhibiting the lowest rank SD (high confidence) among 150 AR inhibitor-anchored drug pairs (400 runs per compound). The red line represents the best fit.

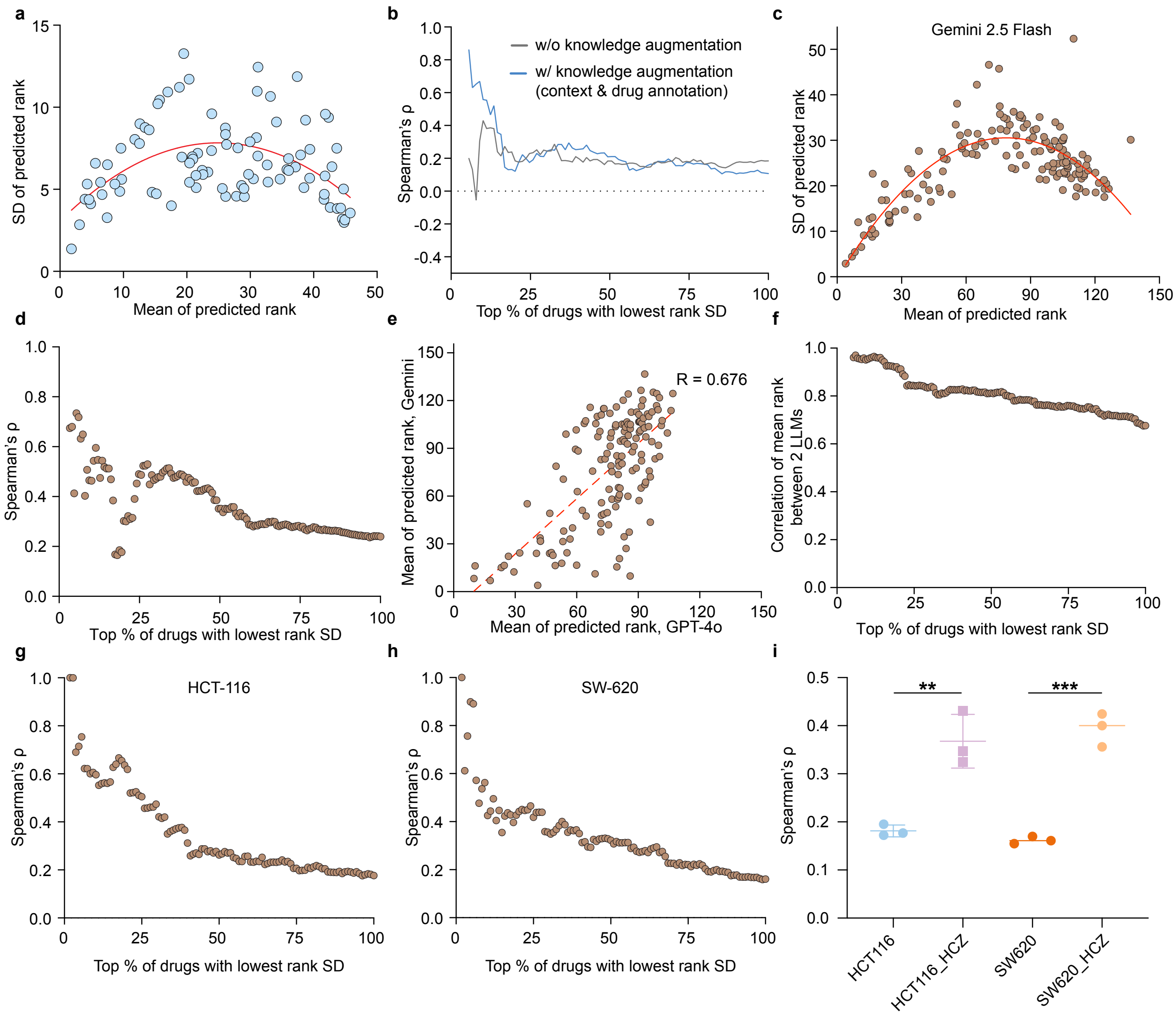

Supplementary Figure 2. MAESTRO demonstrates generalizability across datasets, LLM backends, and cancer types. (**a**) Scatter plot of MAESTRO-predicted rank standard deviation (SD) versus mean rank for 89 abiraterone-anchored drug pairs tested in PC-3 cell line from the DrugComb database using GPT-4o. (**b**) Scatter plot showing Spearman's rank correlation coefficient (ρ) between predicted and experimental rankings as a function of the percentage of drug pairs included (ordered by increasing rank SD) for the dataset shown in **(a)**. The analysis compares model performance with and without knowledge augmentation. (**c**) Scatter plot of MAESTRO-predicted rank SD versus mean rank for 150 AR inhibitor-anchored drug pairs using Gemini 2.5 Flash. Red curves in (**a**) and (**c**) indicate second-order polynomial (quadratic) fits. **(d)** Scatter plot showing Spearman's ρ between predicted and experimental rankings plotted against the percentage of drug pairs included (sorted by ascending rank SD) for the dataset in **(c)**. (**e**) Scatter plot comparing mean ranks predicted by Gemini 2.5 Flash (from panel **(c)**) versus GPT-4o (from **Fig. 1b**) for the identical set of 150 AR inhibitor-anchored drug pairs. The red dashed line represents the linear regression fit. (**f**) Scatter plot showing Spearman's ρ between mean ranks predicted by Gemini 2.5 Flash and GPT-4o as a function of the percentage of drug pairs included, ordered by increasing rank SD to assess the stability of agreement shown in (**e**). (**g-h**) Scatter plots showing Spearman's ρ between rankings predicted by GPT-OSS-20B and experimental rankings as a function of the percentage of drug pairs included (ordered by increasing rank SD) for two datasets from the DrugComb database, each comprising 108 5-fluorouracil (5-FU)-anchored drug pairs tested in HCT-116 (**g**) or SW-620 (**h**) colorectal cancer cells. (**i**) Scatter dot plot comparing Spearman's ρ values calculated using the complete datasets and the high-confidence zone (HCZ), defined as the 25% of drug pairs with the lowest rank SD. Lines indicate the mean ± SD (n = 3). Statistical significance was determined using an unpaired two-tailed Student's t-test; * p < 0.05, ** p < 0.01, *** p < 0.001, **** p < 0.0001; ns, not significant.

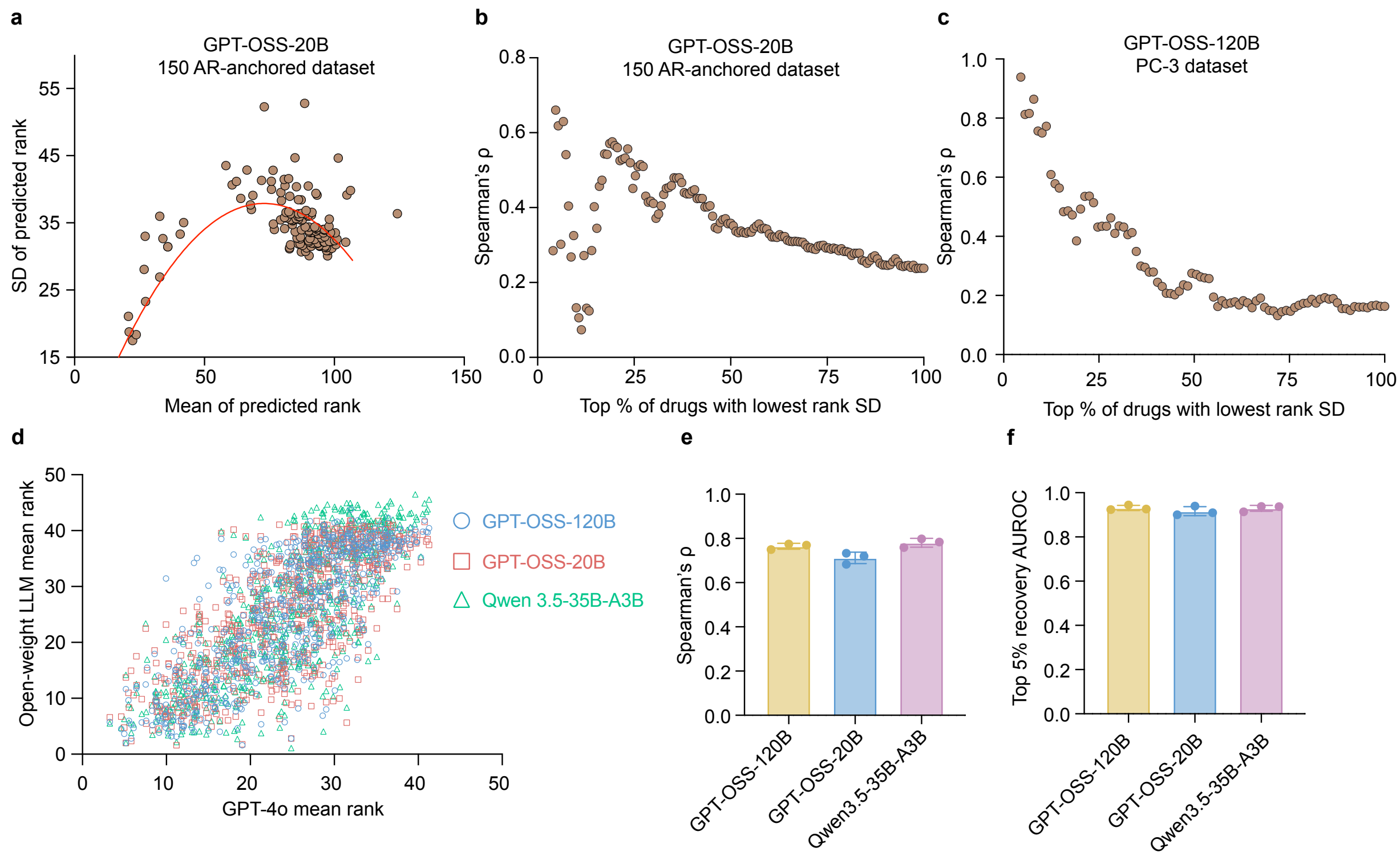

Supplementary Figure 3. Open-weight LLMs reproduce MAESTRO's uncertainty structure, experimental enrichment, and candidate prioritization. (**a**) Predicted rank SD versus mean rank for 150 AR inhibitor-anchored drug pairs analyzed using GPT-OSS-20B. The red line indicates a quadratic fit. (**b-c**) Spearman's ρ between predicted and experimental rankings as a function of the percentage of candidates included, ordered by increasing rank SD, for the AR inhibitor-anchored dataset analyzed with GPT-OSS-20B (**b**) and 89 abiraterone-anchored drug pairs tested in PC-3 cells and analyzed with GPT-OSS-120B (**c**). (**d**) Mean ranks generated by GPT-OSS-120B, GPT-OSS-20B, and Qwen3.5-35B-A3B compared with GPT-4o ranks for one representative set of 700 randomly sampled natural products. (**e-f**) Cross-backend Spearman's ρ (**e**) and AUROC for recovering the GPT-4o-defined top 5% (**f**) across three independently sampled 700-compound datasets. Data are mean ± SD (*n* = 3).

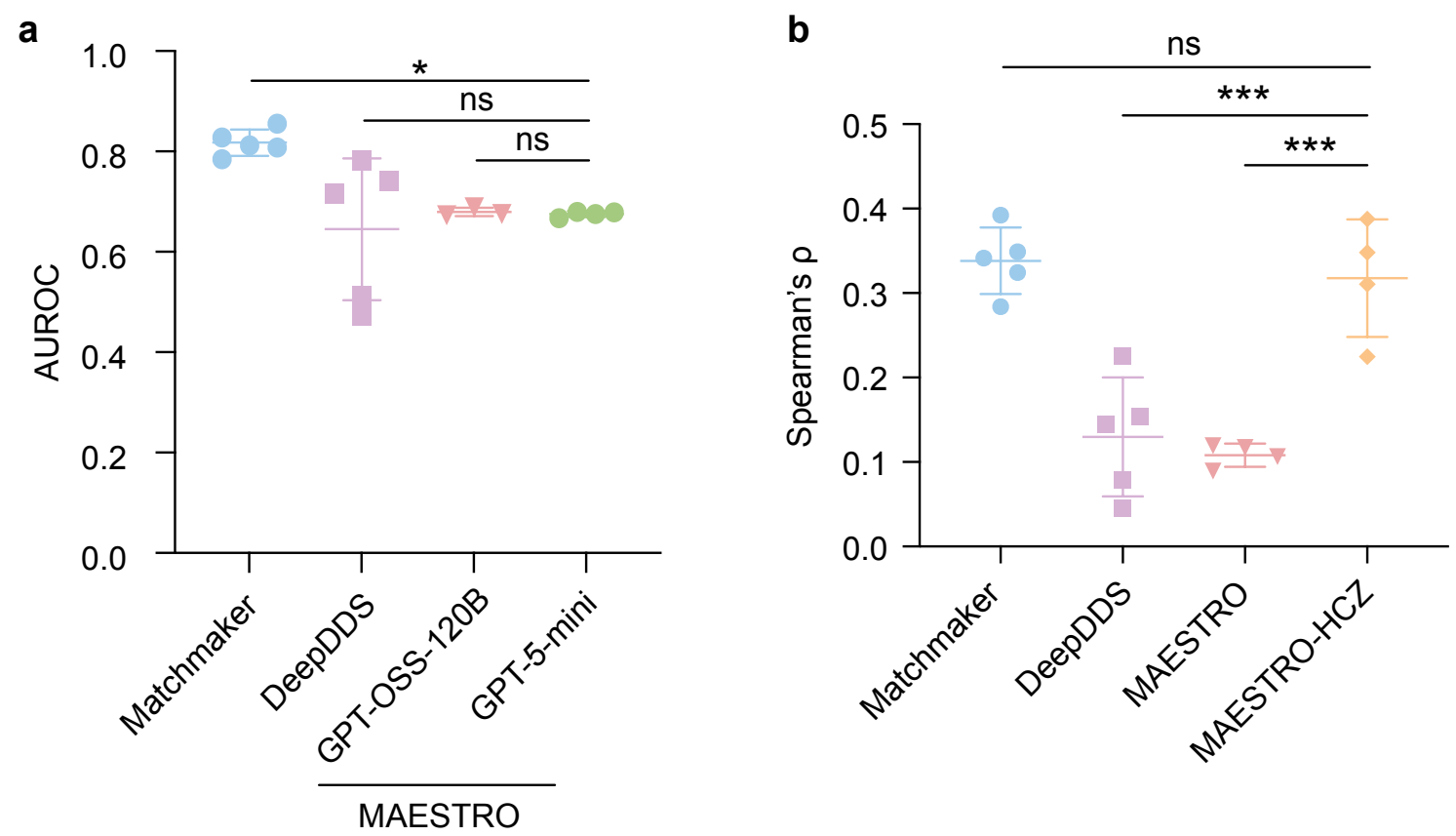

Supplementary Figure 4. MAESTRO achieves predictive performance comparable to trained deep-learning drug-synergy models. (**a**) Scatter dot plot showing the AUROC values for MatchMaker (n = 5), DeepDDS (n = 5), and MAESTRO (GPT-OSS-120B, n = 3; GPT-5 mini, n = 4). (**b**) Spearman's rank correlation between predicted and ground truth rankings for the models, alongside the high-confidence zone subset of MAESTRO (MAESTRO-HCZ, n = 4), generated using GPT-5 mini as the LLM reasoning engine. Both evaluations in (**a**) and (**b**) were based on Bliss synergy scores from a subset of 87 abiraterone-anchored combinations in PC-3 cells. Lines indicate the mean ± SD. Statistical significance was determined using one-way ANOVA followed by Dunnett's multiple comparisons test. (* $p < 0.05$, ** $p < 0.01$, *** $p < 0.001$, **** $p < 0.0001$; ns, not significant).

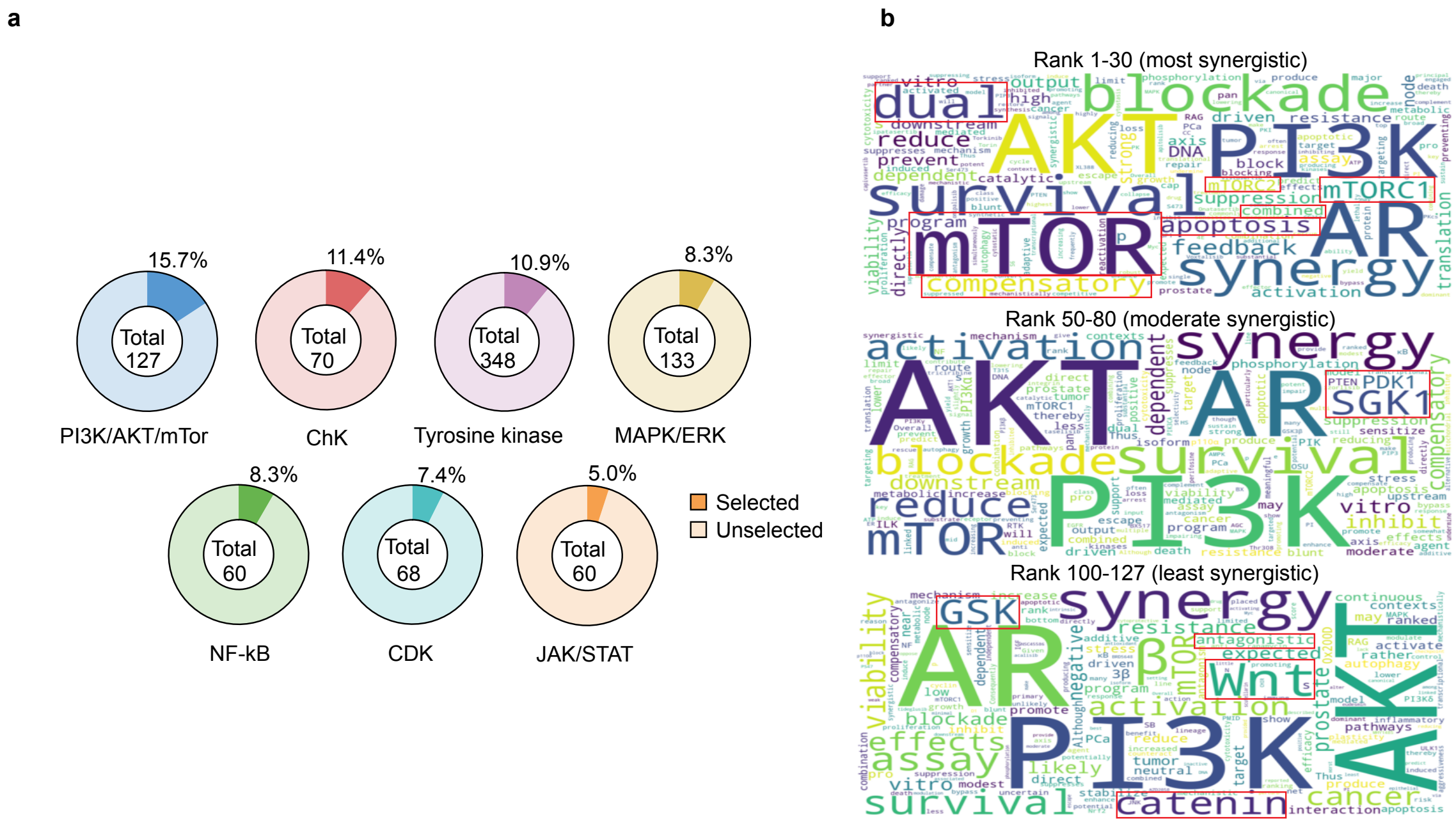

Supplementary Figure 5. Distribution of mechanistic categories and text-mining analysis of MAESTRO predictions. (**a**) Pie charts for each of the 7 mechanistic categories, displaying the total compound count (center) and the percentage of included compounds selected as one of the 95 prioritized candidates. (**b**) Word cloud analysis illustrating the frequency of terms in MAESTRO-generated mechanistic justification (100 runs per inhibitor) for 127 PI3K/AKT/mTOR inhibitors, stratified into three groups based on predicted mean rank. Text size is proportional to the frequency of term occurrence.

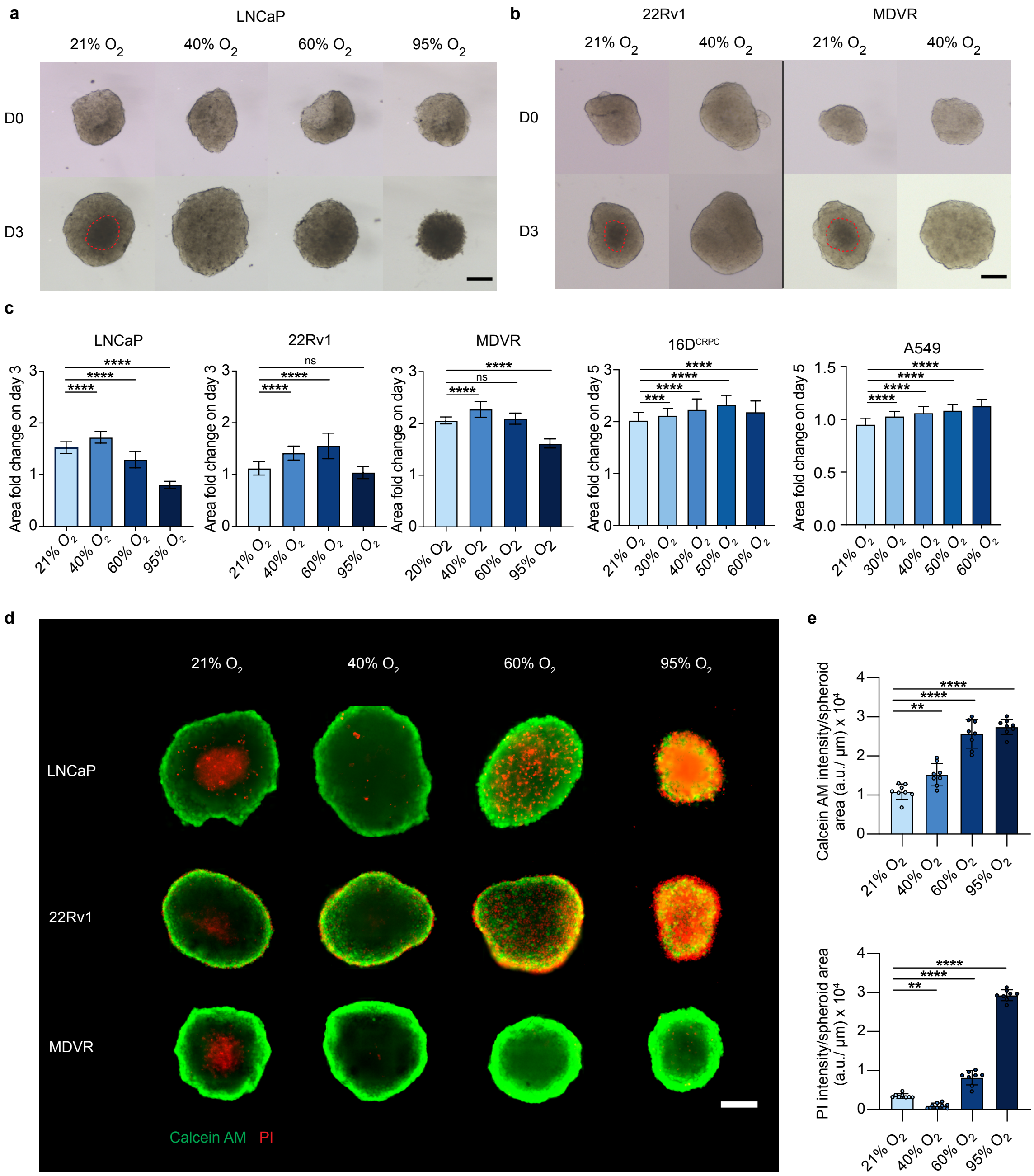

Supplementary Figure 6. Spheroids exhibit robust growth kinetics and cellular viability under optimal oxygen supplementation. (**a-b**) Representative bright-field images of (**a**) LNCaP, (**b**, left) 22Rv1, and (**b**, right) MDVR spheroids cultured under indicated oxygen levels 0 (top) and 3 (bottom) days after spheroid formation. (**c**) Bar charts showing spheroid area fold change at day 3 (LNCaP, 22Rv1, and MDVR) or day 5 ($16D^{CRPC}$ and A549) under indicated oxygen levels in LNCaP (n = 82-106), 22Rv1 (n = 35-49), MDVR (n = 16-26), $16D^{CRPC}$ (n = 75-125), and A549 (n = 37-48) spheroids. (**d**) Representative Live/Dead staining images of LNCaP, 22Rv1, and MDVR spheroids cultured under indicated oxygen levels. Green signal: Calcein AM (live cells); Red signal: Propidium Iodide (PI, dead cells). (**e**) Quantification of integrated Calcein AM (top) and PI (bottom) signal intensity normalized to spheroid area in LNCaP spheroids across different oxygen levels (n = 8). Scale bar, 200 µm. Data are presented as mean ± SD. Statistical significance was determined by one-way ANOVA followed by Dunnett's multiple comparisons test; * $p < 0.05$, ** $p < 0.01$, *** $p < 0.001$, **** $p < 0.0001$, ns: not significant.

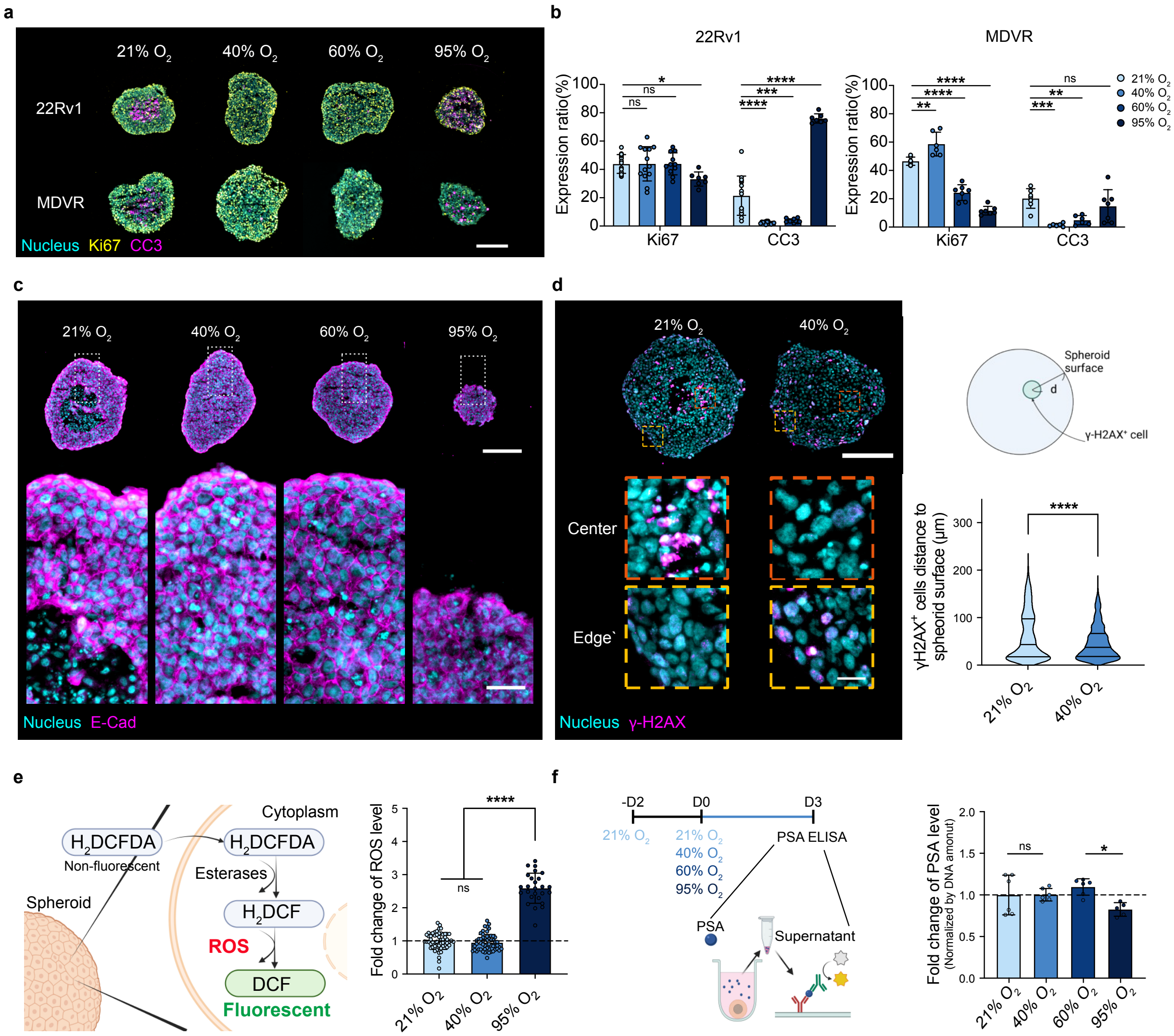

Supplementary Figure 7. Spheroids maintain structural integrity and physiological function under optimal oxygen supplementation. (**a-b**) Representative immunofluorescence images of (**a**) Ki67 (proliferation marker; yellow) and cleaved caspase-3 (CC3; apoptosis marker; magma) in 22Rv1 (*n* =7-15) and MDVR (*n* = 6-7) spheroids cultured under indicated oxygen levels and (**b**) corresponding quantification. Scale bar, 200 µm. (**c**) Representative E-cadherin (E-cad) staining of LNCaP spheroids illustrating cell junction integrity. Bottom panels display magnified views of the regions outlined in white, spanning from the spheroid edge to the spheroid core. Scale bars, 200 µm (top) and 40 µm (bottom). (**d**) Representative immunofluorescence staining of γ-H2AX (DNA double-strand break marker) in LNCaP spheroids cultured under 21% and 40% O_2 (left). Bottom panels show magnified views of the spheroid core and edge regions. Violin plot (right, bottom) quantifies the shortest distance from each γ-H2AX+ cell to the spheroid surface (*n* = 979 cells for 21% O_2; *n* = 675 cells for 40% O_2). Scale bars, 200 µm (top) and 20 µm (bottom). (**e**) Schematic illustrations (left) and corresponding quantification (right) of intracellular ROS levels using H_2DCFDA (*n* = 56 for 21% and 40% O_2; *n* = 26 for 95% O_2). (**f**) Secreted PSA levels quantified via ELISA in LNCaP spheroids on day 3 of spheroid culture (left). Bar charts (right) present values normalized to total DNA content and relative to the 21% O_2 control (*n* = 6). Data are presented as mean ± SD. Statistical significance was determined by one-way ANOVA followed by Dunnett's multiple comparisons test (**b, e, f**) or two-tailed unpaired Student's *t*-test (**d**); * $p < 0.05$, ** $p < 0.01$, *** $p < 0.001$, **** $p < 0.0001$, ns: not significant.

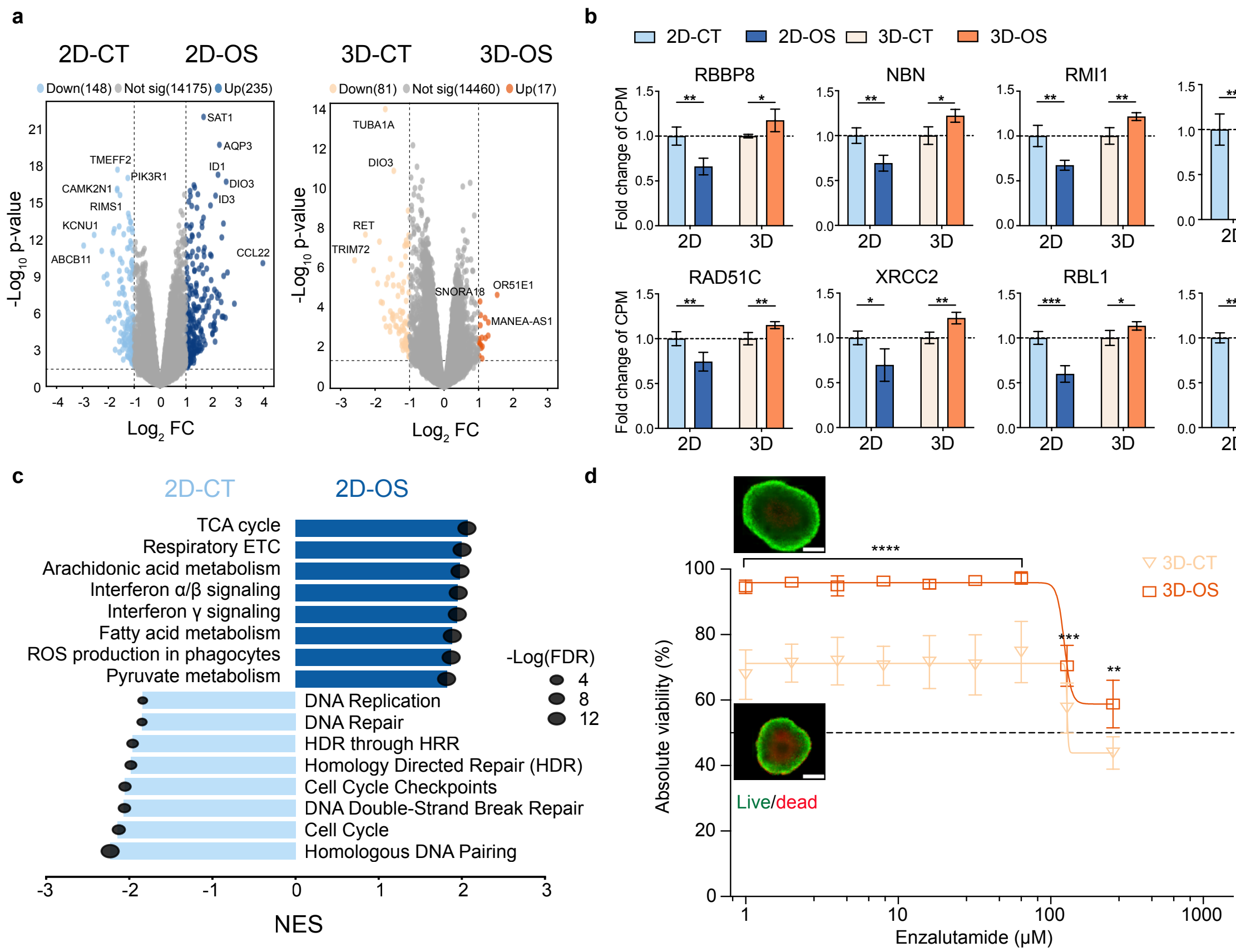

Supplementary Figure 8. Oxygen supplementation modulates transcriptomic profiles and drug sensitivity in prostate cancer spheroids. (**a**) Volcano plots displaying differential gene expression in LNCaP cells comparing 2D 21% O_2 (2D-CT) vs. 2D 40% O_2 (2D-OS) and 3D 21% O_2 (3D-CT) vs. 3D 40% O_2 (3D-OS). Dotted lines indicate significance thresholds ($|log_2FC| \geq 1$, $p < 0.05$; $n = 4$). (**b**) Bar charts showing representative genes related to DNA repair and cell cycle pathways. Oxygen supplementation led to downregulation of these genes in 2D cultures, whereas this effect was reversed in 3D spheroids ($n = 4$). (**c**) Gene Set Enrichment Analysis (GSEA) of upregulated and downregulated pathways in the 2D-CT versus 2D-OS comparison ($n = 4$). (**d**) Enzalutamide dose-response curves plotting absolute viability (from Live/Dead staining) for 22Rv1 spheroids in 3D-CT and 3D-OS groups. Insets display representative images of untreated controls at Day 3 ($n = 6$). Scale bar, 200 µm. Data are presented as mean ± SD. Statistical significance was determined by two-tailed unpaired Student's *t*-test; * $p < 0.05$, ** $p < 0.01$, *** $p < 0.001$, **** $p < 0.0001$, ns: not significant.

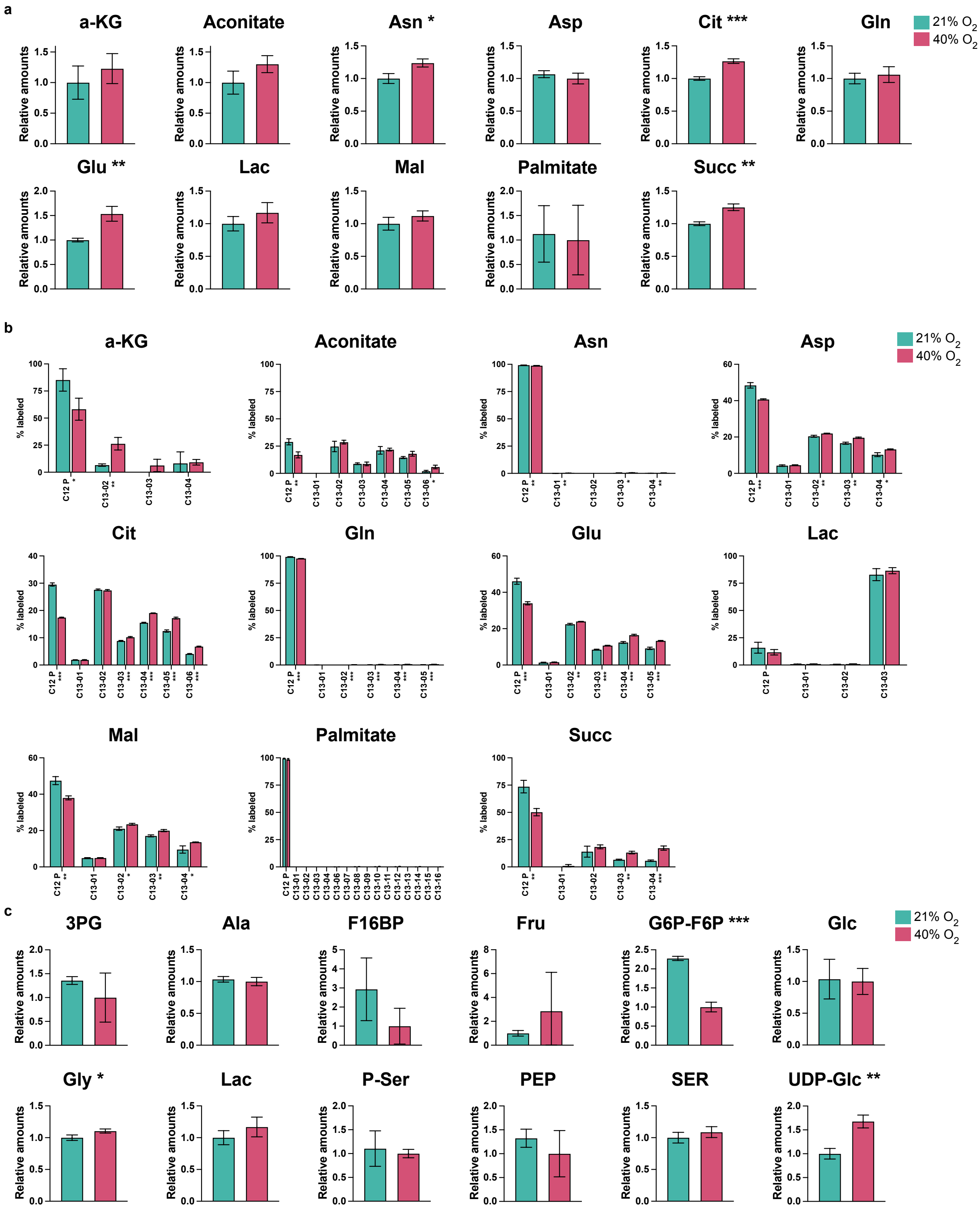

a
a-KG
Aconitate
Asn *
Asp
Cit ***
Gln
Glu **
Lac
Mal
Palmitate
Succ **
Relative amounts
21% O_2
40% O_2
b
a-KG
Aconitate
Asn
Asp
Cit
Gln
Glu
Lac
Mal
Palmitate
Succ
% labeled
21% O_2
40% O_2
c
3PG
Ala
F16BP
Fru
G6P-F6P ***
Glc
Gly *
Lac
P-Ser
PEP
SER
UDP-Glc **
Relative amounts
21% O_2
40% O_2

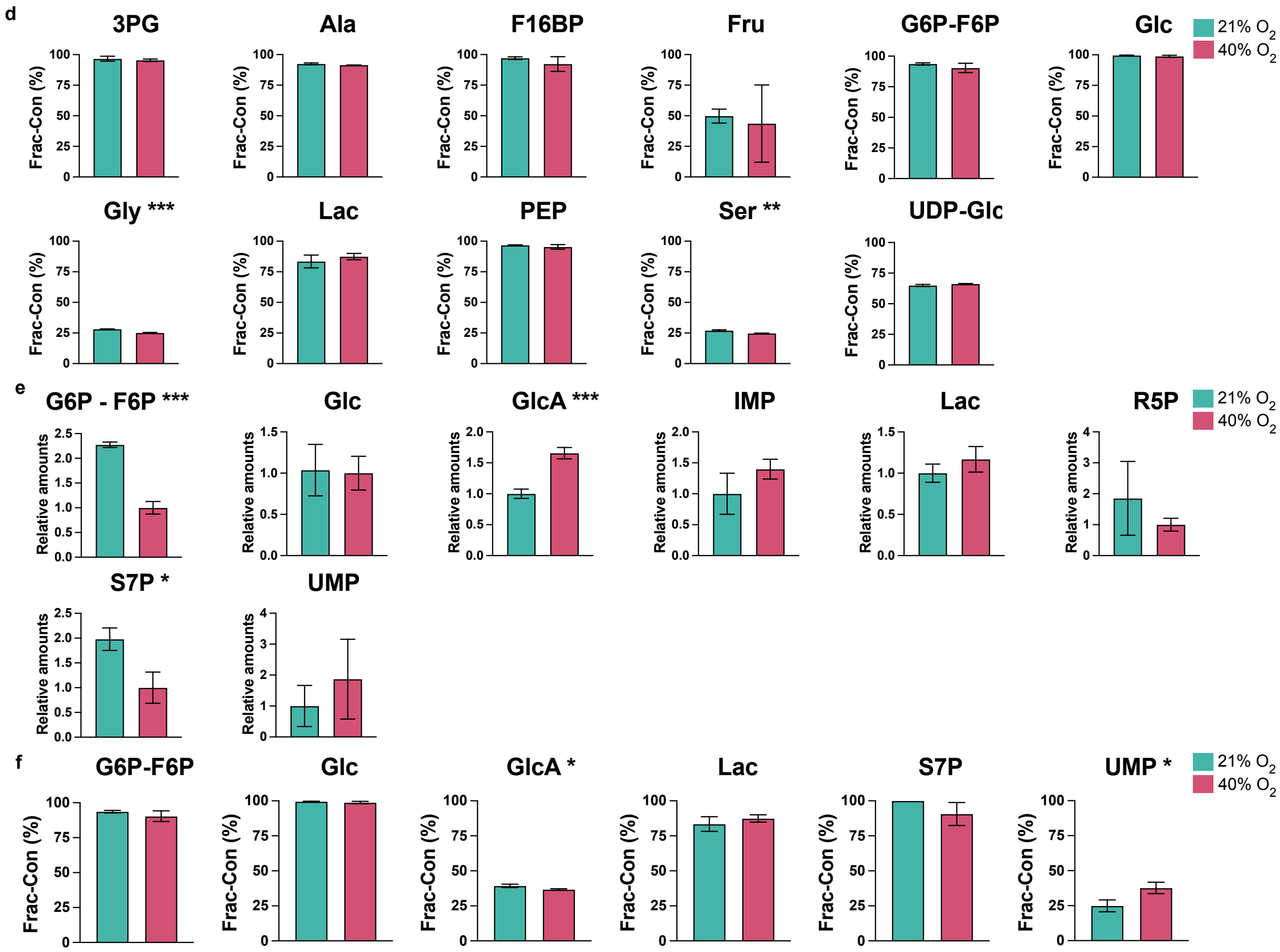

Supplementary Figure 9. Metabolomic profiling reveals that oxygen supplementation promotes glucose entry into the TCA cycle without broadly altering glycolysis or the pentose phosphate pathway. (**a-b**) Relative amounts **(a)** and isotopologue distribution **(b)** of TCA cycle metabolites in LNCaP spheroids cultured under indicated oxygen levels. (**c-d**) Relative amounts **(c)** and fractional contribution **(d)** to glycolytic metabolites in LNCaP spheroids cultured under indicated oxygen levels. **(e-f)** Relative amounts **(e)** and fractional contribution **(f)** to pentose phosphate pathway (PPP) metabolites in LNCaP spheroids cultured under indicated oxygen levels. Spheroids were labeled with D-[U-$^{13}C_6$] glucose for 24 h prior to analysis. Data are presented as mean ± SD (n = 3). Statistical significance was determined by two-tailed unpaired Student's t-test; * $p < 0.05$, ** $p < 0.01$, *** $p < 0.001$, **** $p < 0.0001$, ns: not significant. 3PG, 3-Phosphoglycerate; Ala, Alanine; F16BP, Fructose-1,6-bisphosphate; Fru, Fructose; G6P-F6P, Hexose phosphate; Glc, Glucose; Gly, Glycine; Lac, Lactate; P-Ser, Phosphoserine; PEP, Phosphoenolpyruvate; Ser, Serine; UDP-Glc, UDP-glucose; α-KG, α -Ketoglutarate; Asn, Asparagine; Asp, Aspartate; Cit, Citrate; Gln, Glutamine; Glu, Glutamate; Mal, Malate; Succ, Succinate; GlcA, Gluconic acid; IMP, Inosine monophosphate; R5P, Ribose-5-phosphate; S7P, Sedoheptulose-7-phosphate.

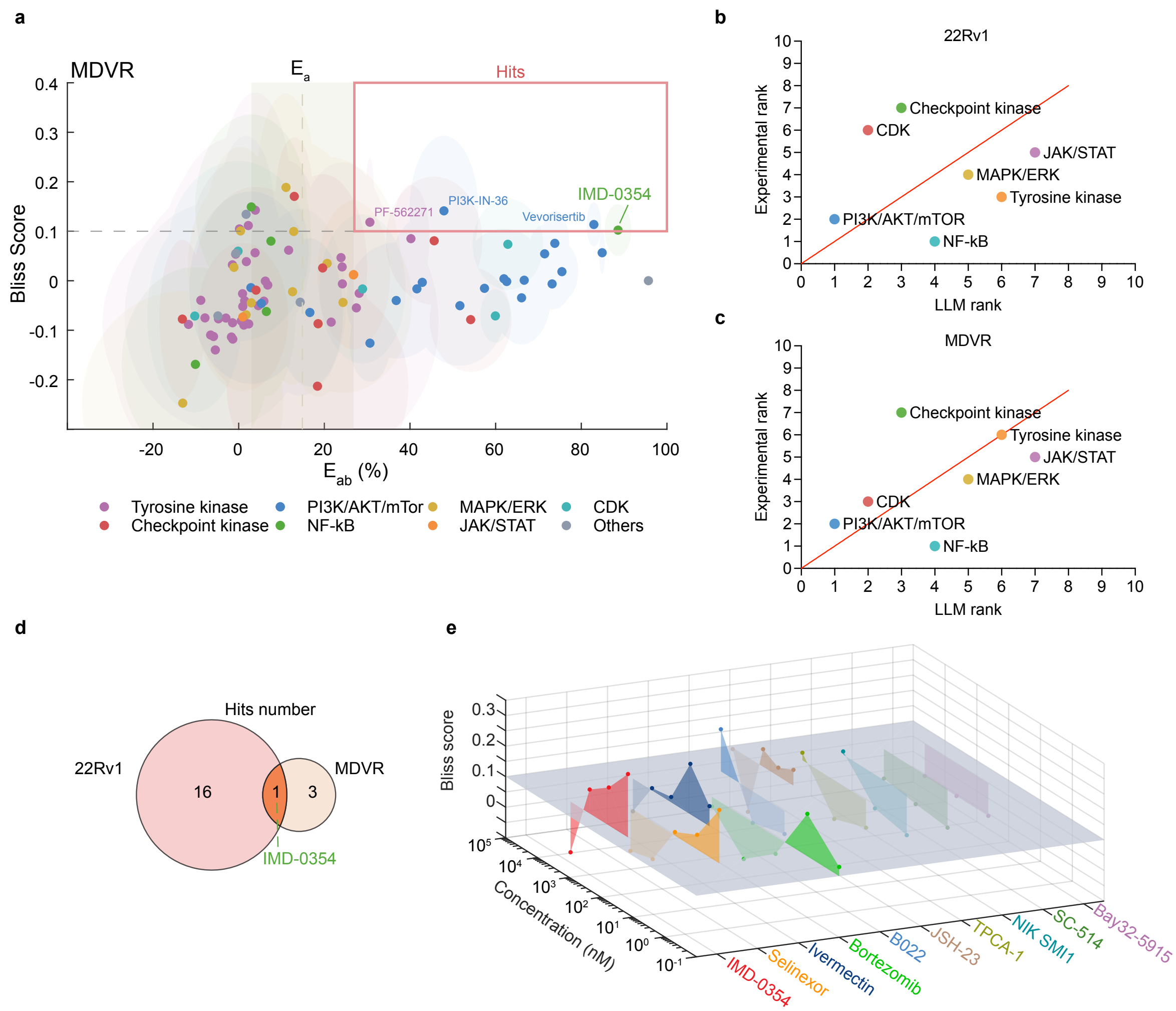

Supplementary Figure 10. In vitro screening validates the pathway-specific predictive accuracy of MAESTRO-prioritized PKIs. (**a**) Scatter plot of Bliss score versus E_{ab} for 95 PKIs tested in oxygenated MDVR spheroids. Hits (red boxed region) are defined as compounds with E_{ab} greater than enzalutamide monotherapy (E_a) and a Bliss score > 0.1. Error ellipses denote the SD (n = 4) for E_{ab} (x-axis) and Bliss scores (y-axis). (**b-c**) Scatter plots displaying the relationship between experimental rank and category-averaged MAESTRO rank (LLM rank) across 7 mechanistic categories in (**b**) 22Rv1 and (**c**) MDVR spheroids cultured under 40% O_2. (**d**) Venn diagram displaying hit numbers identified in oxygenated 22Rv1 and MDVR spheroids and their shared hit, IMD-0354. (**e**) Three-dimensional plot displaying Bliss scores for 10 NF-κB inhibitors across multiple concentrations.

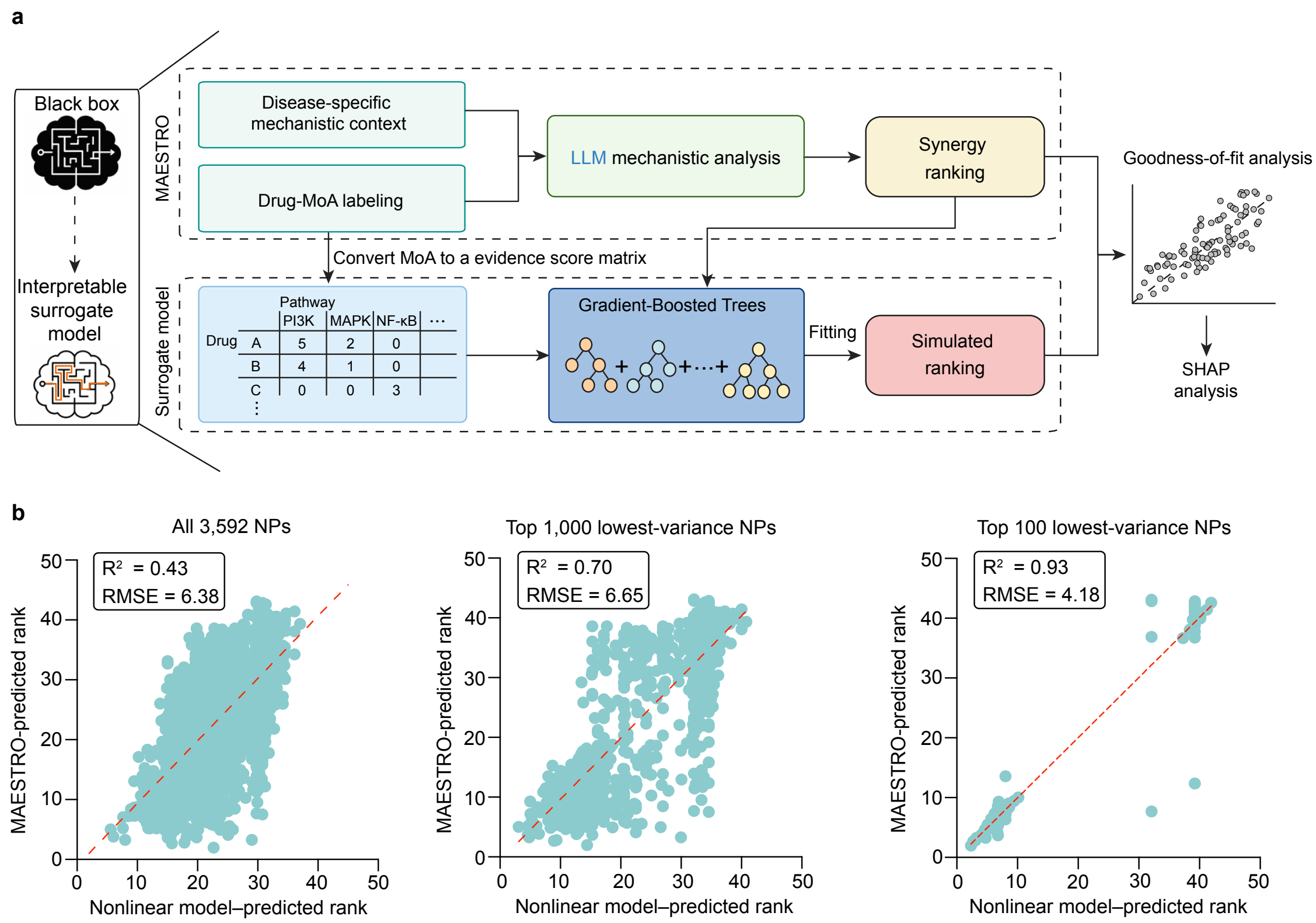

Supplementary Figure 11. GBT surrogate model captures MAESTRO's pathway-based synergy prediction logic. (a) Schematic illustration of the workflow for deconvolving MAESTRO's prediction logic. The diagram depicts the conversion of MAESTRO's input, Drug-MoA annotation, to evidence scores across 12 pathways, which subsequently serve as input features for the GBT surrogate model to predict synergy ranks and the downstream SHAP (SHapley Additive exPlanations) analysis. **(b)** Correlation plots illustrating the MAESTRO-predicted mean rank versus the nonlinear GBT model-predicted rank. Panels represent the full dataset (left), top 1,000 candidates (middle), and top 100 lowest-variance (high confidence) candidates (right). Red dashed lines indicate linear regression fits.

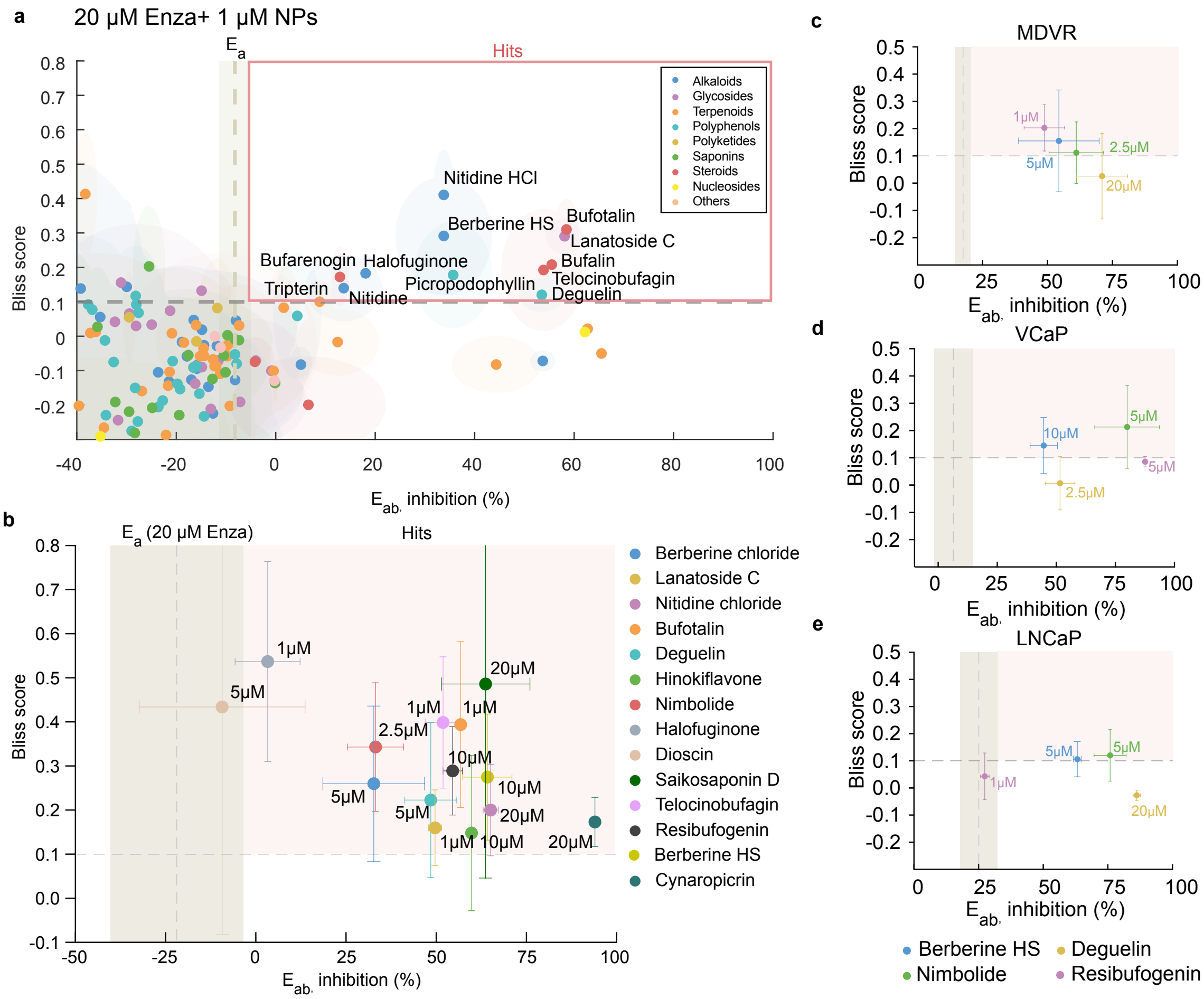

Supplementary Figure 12. Multi-stage screening identifies natural products with reproducible enzalutamide synergy across multiple prostate cancer models. (**a**) Experimental screening of 150 prioritized candidates (1 μM) combined with Enzalutamide (20 μM) in 22Rv1 spheroids. Scatter plot shows combination efficacy (E_{ab}) versus Bliss scores. E_{ab} was calculated as the inhibition rate (%) relative to the untreated group. Hits (red box): E_{ab} > enzalutamide monotherapy (E_a) and Bliss > 0.1. Error ellipses: SD (*n* = 4). Vertical line/shade: mean ± SD of E_a (*n* = 20). (**b**) Validation of 14 top-tier candidates showing Bliss score versus E_{ab}. Labels indicate the concentration yielding the highest Bliss score for each compound (*n* = 4). (**c-e**) Synergy validation of berberine hydrogen sulphate, deguelin, nimbolide, and resibufogenin across MDVR **(c)**, VCaP **(d)**, and LNCaP **(e)** spheroids (*n* = 8). In (**a–e**), the vertical dashed lines and shaded areas represent the mean ± SD of enzalutamide monotherapy efficacy (E_a). Data are presented as mean ± SD.

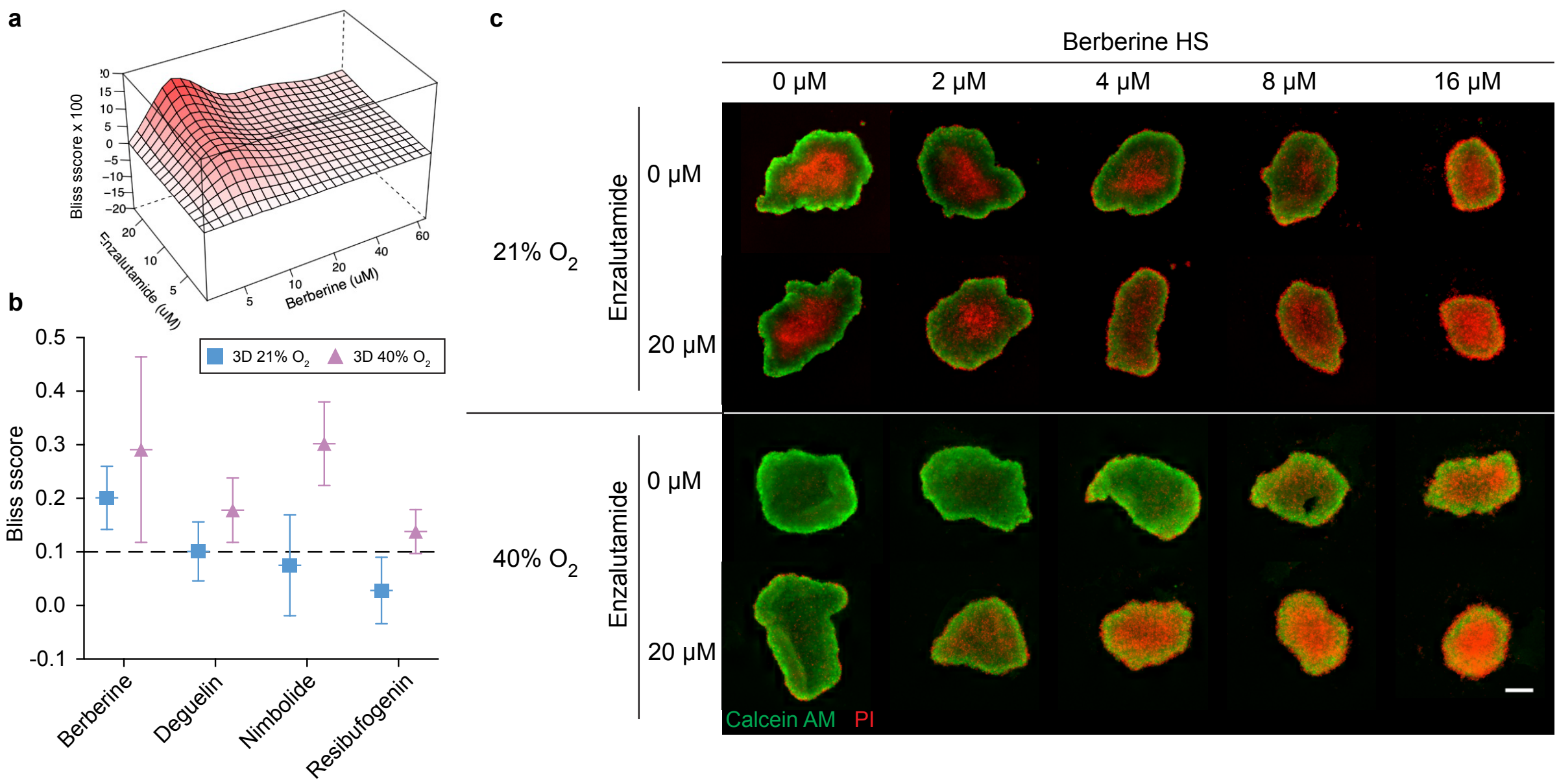

Supplementary Figure 13. Necrotic cores may compromise synergy detection. (**a**) three-dimensional synergy plot of the berberine–enzalutamide combination (*n* = 12) on 2D 22Rv1 cell culture model. **(b)** Bliss scores of berberine, deguelin, nimbolide, and resibufogenin in combination with enzalutamide in standard 3D (21% O_2) and oxygen-supplemented 3D (40% O_2) 22Rv1 models (*n* = 4). Data are presented as mean ± SD. **(c)** Representative Live/Dead staining images of berberine-enzalutamide co-treatment on 22Rv1 spheroids cultured under indicated oxygen levels. Green signal: Calcein AM (live cells); Red signal: propidium Iodide (PI, dead cells); Scale bar, 200 µm.

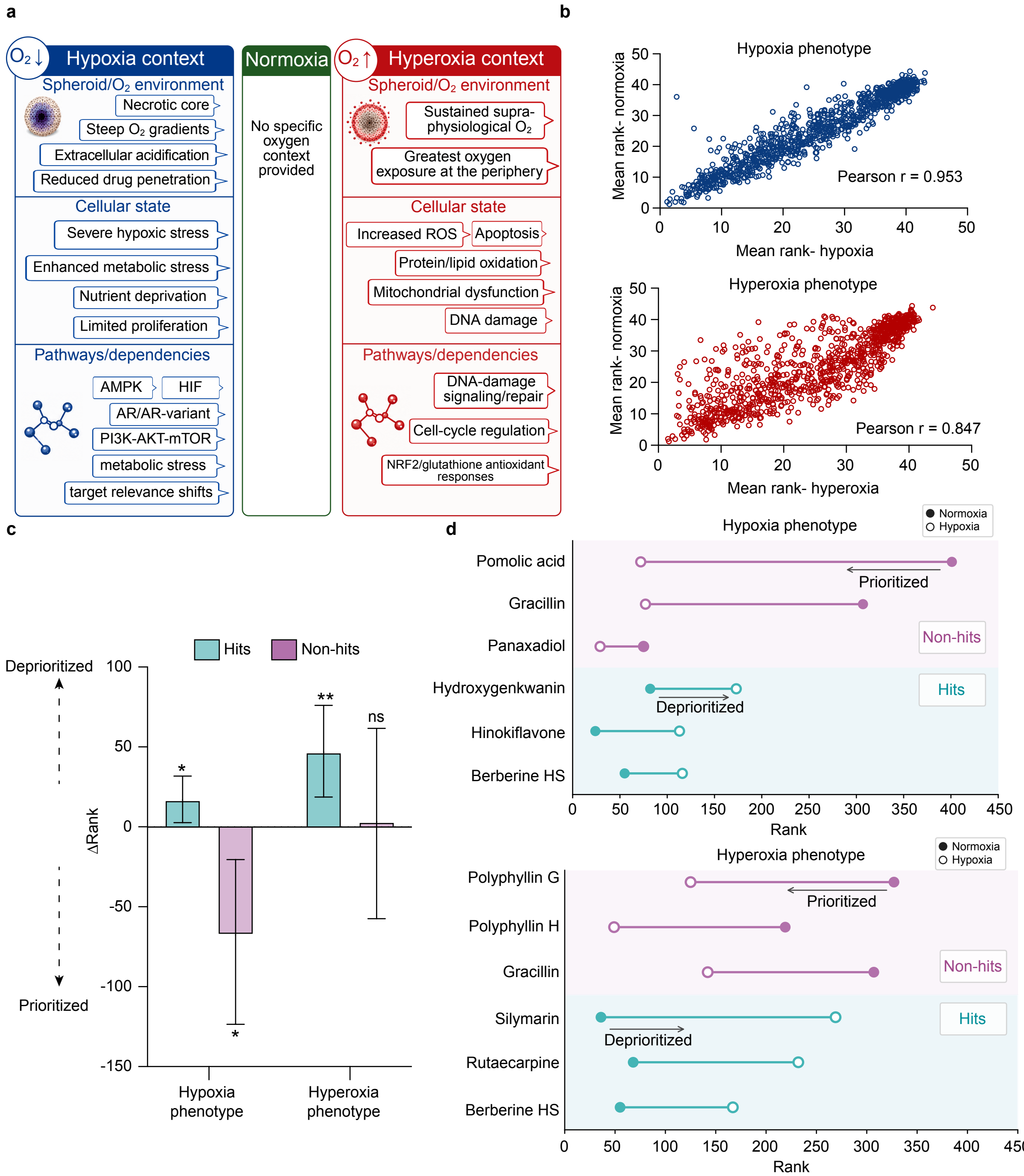

Supplementary Figure 14. Imposing severe hypoxia or hyperoxia contexts reduces the prioritization of experimentally identified hits. (**a**) Schematic of the physiological assumptions added to MAESTRO prompts for drug-synergy ranking. The baseline prompt contained no explicit oxygen-related assumptions. The hypoxia and hyperoxia prompts incorporated the indicated assumptions regarding the spheroid microenvironment, cellular state, and oxygen-associated pathway dependencies. (**b**) Scatter plots comparing mean ranks generated using the baseline prompt with those generated under the hypoxia (top) or hyperoxia (bottom) context for 1,000 natural products, comprising 150 experimentally tested candidates and 850 randomly sampled natural products. Predictions were generated using GPT-OSS-20B. Pearson correlation coefficients are shown. (**c**) Changes in predicted rank among experimentally classified hits and non-hits after adding the hypoxia or hyperoxia context. ΔRank was calculated as the context-conditioned rank minus the corresponding baseline rank; positive values indicate deprioritization and

negative values indicate prioritization. Hits were defined as Bliss scores > 0.10 (n = 42), and non-hits as Bliss scores < −0.10 (n = 15). Data are presented as means with 95% percentile bootstrap confidence intervals generated from 20,000 resamples. Statistical significance relative to zero was assessed using two-sided null-centered bootstrap tests with Benjamini–Hochberg correction across the four prespecified comparisons; * $q < 0.05$, ** $q < 0.01$; ns, not significant. (**d**) Representative rank shifts for individual experimental hits and non-hits after incorporating hypoxia (top) or hyperoxia (bottom) assumptions. Filled circles indicate baseline ranks, and open circles indicate context-conditioned ranks.

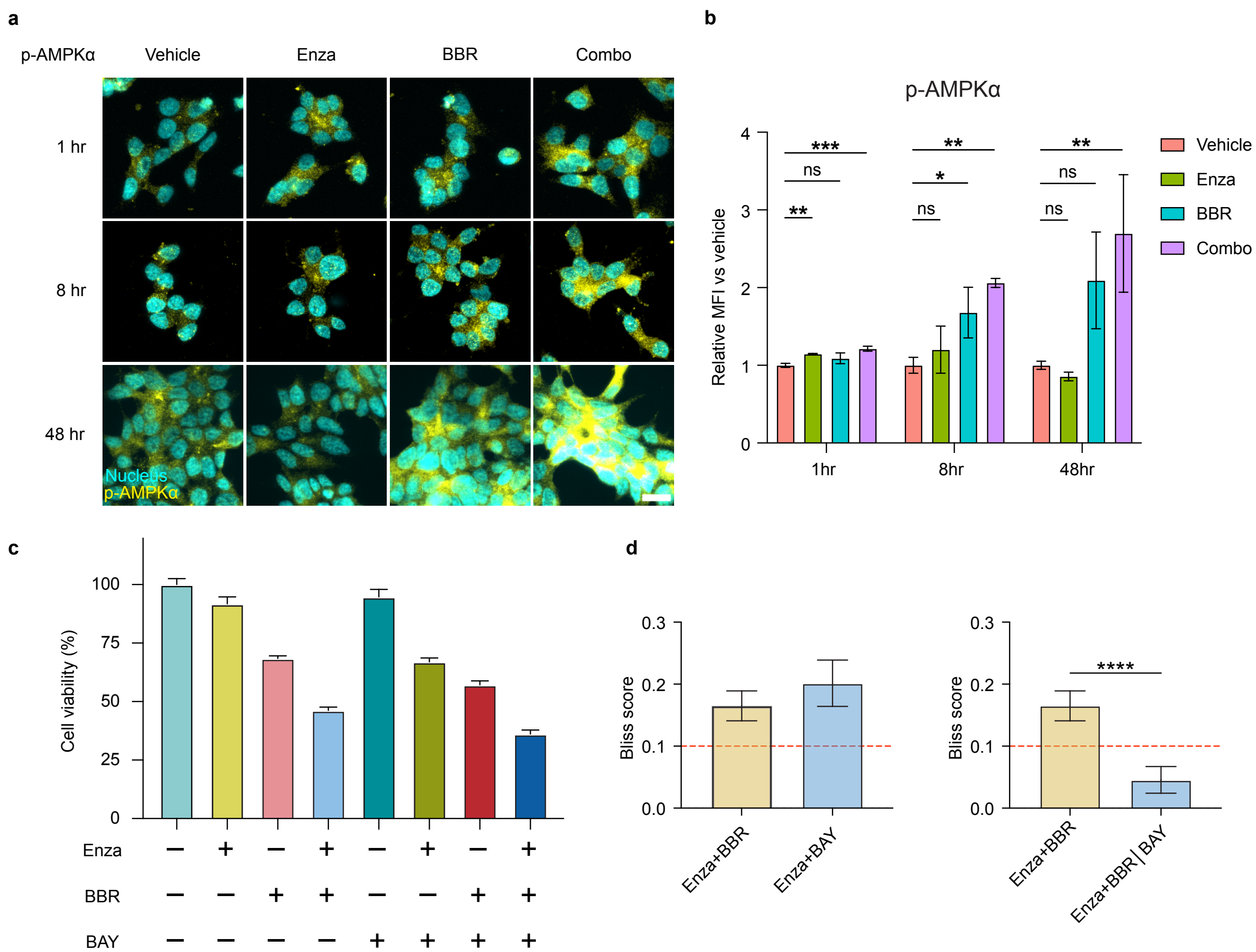

Supplementary Figure 15. Time-course and pharmacological inhibition studies support AMPK involvement in berberine–enzalutamide synergy. (**a–b**) Representative immunofluorescence images (**a**) and corresponding quantification (**b**) of phospho-AMPKα (p-AMPKα; yellow) in 22Rv1 cells treated with vehicle, 20 μM enzalutamide (Enza), 5 μM berberine (BBR), or their combination (Combo) for 1, 8, or 48 h. Nuclei are shown in cyan. Background fluorescence was subtracted from the p-AMPKα mean fluorescence intensity (MFI). The background-corrected MFI was expressed as fold change relative to the corresponding vehicle group at each time point. Scale bar, 20 μm. Data are presented as mean ± SD ($n = 3$). Statistical significance was determined using one-way ANOVAs at each time point, followed by Dunnett's multiple-comparisons test comparing each treatment with the corresponding vehicle control; * $p < 0.05$, ** $p < 0.01$, *** $p < 0.001$, **** $p < 0.0001$; ns, not significant. (**c–d**) Bar charts showing results from a three-drug combination experiment with the AMPK inhibitor BAY-3827 in 22Rv1 cells. (**c**) Relative cell viability following 72-h treatment with the indicated combinations of enzalutamide (20 μM), berberine (5 μM), and BAY-3827 (0.5 μM). Data are presented as mean ± SD ($n = 6$). (**d**) Bliss scores for the enzalutamide–berberine, enzalutamide–BAY-3827 combinations (left), and the comparison of the enzalutamide–berberine Bliss score in the absence of BAY-3827 with the conditional enzalutamide–berberine Bliss score in the BAY-3827 background (right). Error bars indicate 95% percentile bootstrap confidence intervals generated from 20,000 resamples. BAY-3827 attenuated the enzalutamide–berberine interaction (ΔBliss = -0.120; 95% bootstrap CI, -0.152 to -0.087; **** *pboot* < 0.0001). Red dashed lines in (**d**) indicate the Bliss synergy threshold of 0.1.

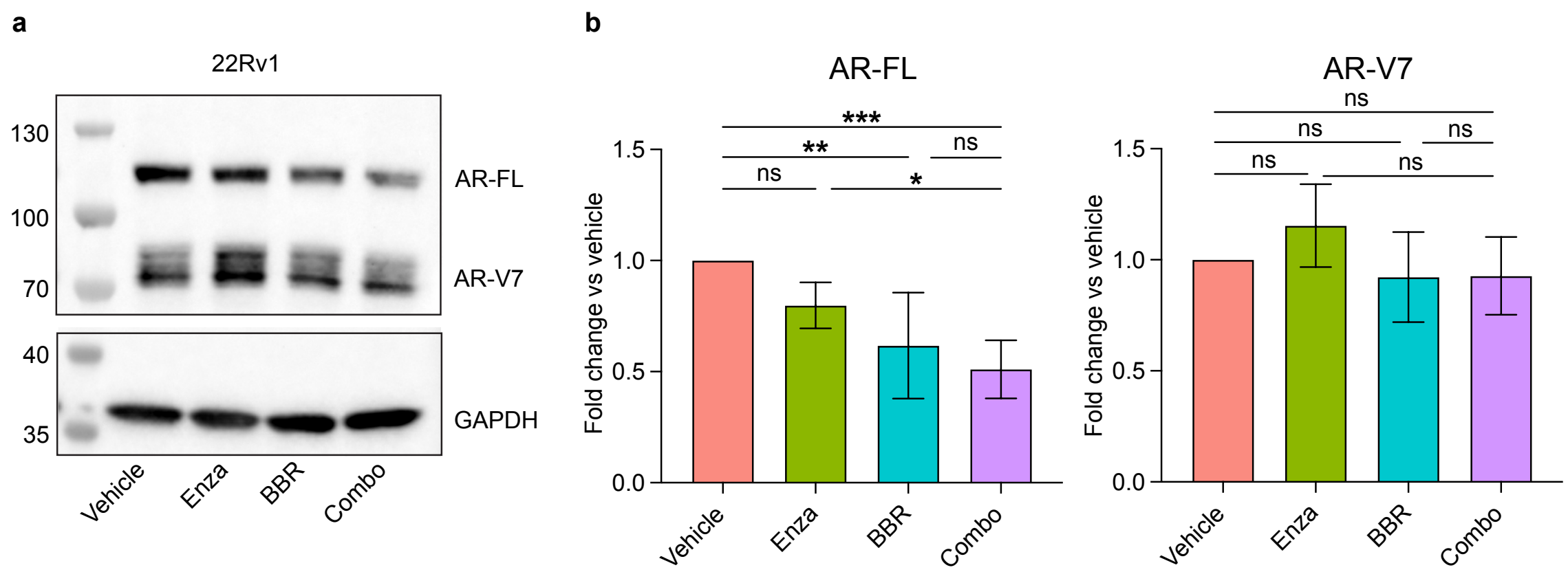

Supplementary Figure 16. Berberine-containing treatments reduce AR-FL protein abundance without altering AR-V7 in 22Rv1 cells. (**a**) Representative immunoblot images of AR-FL, AR-V7, and GAPDH in 22Rv1 cells treated with vehicle, 20 µM enzalutamide (Enza), 5 µM berberine (BBR), or their combination for 24 h. Molecular masses are indicated in kDa. (**b**) Densitometric quantification of AR-FL and AR-V7 normalized to GAPDH and expressed relative to vehicle. Data are mean ± SD (n = 5). Statistical significance was determined by one-way ANOVA followed by Tukey's multiple-comparisons test; * $p < 0.05$, ** $p < 0.01$, *** $p < 0.001$; ns, not significant.

Supplementary Text. Complete context prompts used for the oxygenation-context sensitivity analysis.

Hypoxia context prompt:
The in vitro drug experiments are performed using large 3D tumor spheroids, approximately 500–600 μm in diameter, that develop a pronounced hypoxia-induced necrotic core. Because of steep oxygen, nutrient, metabolite, and drug-concentration gradients across the spheroid, cells in the inner regions are exposed to severe hypoxic stress, nutrient deprivation, extracellular acidification, accumulation of metabolic waste products, reduced drug penetration, and limited proliferative activity. These conditions may generate cellular states that differ substantially from those observed in well-oxygenated, actively proliferating 2D monolayer cultures. When predicting synergy with Enzalutamide, explicitly evaluate how the microenvironment of the spheroid alters the baseline biological state of the tumor cells before assessing the activity of each candidate compound. Consider hypoxia-associated changes in signaling pathways, transcriptional programs, metabolic dependencies, cellular stress responses, cell-cycle progression, survival mechanisms, and drug sensitivity. Relevant processes may include, but are not limited to, HIF-dependent signaling, androgen receptor and AR-variant signaling, PI3K–AKT–mTOR activity, AMPK-mediated energy-stress responses, glycolytic and mitochondrial metabolism, reactive oxygen species regulation, DNA-damage responses, apoptosis, autophagy, unfolded-protein responses, inflammatory signaling, epithelial–mesenchymal plasticity, stem-like cellular states, and mechanisms of treatment resistance. Assess whether the molecular target and mechanism of action of each compound remain biologically active, become more important, or become less relevant under severe hypoxia and nutrient limitation. Determine whether the compound preferentially affects proliferating cells, metabolically active cells, quiescent cells, hypoxia-adapted cells, or cells located near the necrotic boundary. Compounds that depend strongly on cell division, oxygen-dependent chemistry, mitochondrial respiration, or efficient tissue penetration may exhibit reduced activity in the spheroid core. In contrast, compounds targeting hypoxia-induced survival pathways, metabolic stress adaptation, redox homeostasis, autophagy, anti-apoptotic signaling, or other dependencies created by the hypoxic microenvironment may retain or gain activity. Also consider whether hypoxia changes the expression, activation state, accessibility, or functional importance of the drug target. Evaluate possible effects of altered cellular uptake, intracellular accumulation, efflux transport, chemical stability, oxygen-dependent activation, and diffusion through the spheroid. Distinguish reduced pharmacological exposure caused by limited penetration from reduced biological sensitivity caused by pathway adaptation, quiescence, or target downregulation. For the predicted interaction with Enzalutamide, determine whether the candidate compound is expected to reinforce androgen-receptor inhibition, overcome hypoxia-associated resistance to Enzalutamide, target a compensatory survival pathway activated after androgen-receptor blockade, or instead lose effectiveness because the hypoxic cells are less dependent on androgen-receptor signaling or are less proliferative. Consider whether Enzalutamide and the candidate compound act on complementary vulnerabilities, redundant pathways, or potentially antagonistic cellular programs under these conditions. Incorporate spatial heterogeneity within the spheroid into the prediction. The final measured response represents the combined effects on well-oxygenated peripheral cells, moderately hypoxic intermediate cells, severely hypoxic or quiescent inner cells, and cells adjacent to the necrotic core. A compound may therefore remain highly active in the outer proliferative region while showing limited activity in the core, or it may preferentially affect stress-adapted cells in the inner region. Evaluate whether the expected overall spheroid response is likely to be dominated by one of these cellular compartments. For each compound, determine whether the hypoxic and necrotic spheroid environment is expected to increase, decrease, or have minimal effect on its individual activity and its synergistic interaction with Enzalutamide. Estimate the likely magnitude of this effect as small, moderate, or substantial, based on the strength and directness of the mechanistic relationship. Incorporate these predicted physiological effects into the mechanistic reasoning and final drug ranking. Do not assume that hypoxia uniformly decreases drug effectiveness; adjust the ranking only when there is a plausible mechanistic connection between the spheroid-associated physiological state and the compound's mechanism of action.

Hyperoxia context prompt:
The in vitro drug experiments are performed using large 3D tumor spheroids, approximately 500–600 μm in diameter, exposed to sustained supraphysiological oxygen conditions sufficient to produce true hyperoxic stress. Under these conditions, excessive oxygen exposure may itself become a source of cellular stress and injury rather than merely improving oxygen availability or reducing diffusion-limited necrosis.
When predicting synergy with Enzalutamide, explicitly evaluate how hyperoxia may alter the baseline biological state of the tumor cells before assessing the interaction between Enzalutamide and each candidate compound. Consider whether reported hyperoxia-associated changes affect the compound's molecular target, mechanism of

action, or relevant cellular dependency, and incorporate these effects into the mechanistic reasoning and final synergy ranking when relevant.

Hyperoxia has been reported to increase reactive oxygen species and oxidative damage, although the magnitude varies with cell type, exposure duration, metabolic state, and antioxidant capacity. Reported responses include oxidation of proteins and membrane lipids, mitochondrial dysfunction, mitochondrial and nuclear DNA damage, activation of DNA-damage checkpoints, inhibition of DNA synthesis, cell-cycle arrest, reduced proliferation or loss of proliferative capacity, and apoptosis. Sustained hyperoxia may also activate compensatory antioxidant responses, including NRF2-dependent transcription and changes in glutathione-associated defenses, although the magnitude and effectiveness of these responses vary across cell types and exposure conditions. Increased oxygen availability should therefore not automatically be interpreted as increased mitochondrial respiration or metabolic activity, because prolonged hyperoxia may instead impair mitochondrial function and bioenergetic capacity.

Assess whether the candidate compound directly affects oxidative stress, antioxidant defenses, mitochondrial function, DNA-damage signaling, DNA repair, or cell-cycle progression and whether these effects could modify its interaction with Enzalutamide. Consider whether hyperoxia and the compound create complementary vulnerabilities, converge redundantly on the same injury, or activate opposing cytotoxic and protective responses.

Because the spheroids are large, hyperoxic exposure may remain spatially heterogeneous. Peripheral cells are expected to experience the greatest increase in oxygen exposure, whereas oxygen availability may remain progressively lower toward the center. The final measured response therefore combines effects on strongly hyperoxia-exposed peripheral cells, metabolically active intermediate cells, and less-oxygenated inner cells. Determine whether the relevant drug mechanism is likely to affect a limited cellular compartment or multiple regions of the spheroid and whether this spatial distribution could materially influence the overall combination response.

For the predicted interaction with Enzalutamide, consider whether the candidate compound affects a survival or damage-response mechanism that becomes relevant when androgen-receptor inhibition occurs in the presence of hyperoxic stress. Determine whether the two treatments act on complementary vulnerabilities, redundant pathways, or potentially antagonistic cellular programs.

Give greater weight to evidence directly comparing hyperoxic and non-hyperoxic conditions, particularly in cancer cells or comparable 3D models. For each compound, determine whether the available evidence suggests that hyperoxia may alter its synergistic interaction with Enzalutamide and estimate the likely direction and magnitude of the effect as small, moderate, or substantial. Incorporate these considerations into the final ranking according to the strength, directness, and biological relevance of the evidence, without assuming that hyperoxia uniformly increases or decreases drug synergy.
